# High‐precision magnetoencephalography for reconstructing amygdalar and hippocampal oscillations during prediction of safety and threat

**DOI:** 10.1002/hbm.24689

**Published:** 2019-06-30

**Authors:** Athina Tzovara, Sofie S. Meyer, James J. Bonaiuto, Aslan Abivardi, Raymond J. Dolan, Gareth R. Barnes, Dominik R. Bach

**Affiliations:** ^1^ Department of Psychiatry, Psychotherapy, and Psychosomatics University of Zurich Zurich Switzerland; ^2^ Neuroscience Centre Zurich University of Zurich Zurich Switzerland; ^3^ Wellcome Centre for Human Neuroimaging Institute of Neurology, University College London London United Kingdom; ^4^ Helen Wills Neuroscience Institute, University of California Berkeley California; ^5^ UCL Institute of Cognitive Neuroscience, University College London London United Kingdom; ^6^ Max Planck UCL Centre for Computational Psychiatry and Ageing Research University College London London United Kingdom

**Keywords:** amygdala, hippocampus, MEG, source reconstruction, theta oscillations, threat conditioning

## Abstract

Learning to associate neutral with aversive events in rodents is thought to depend on hippocampal and amygdala oscillations. In humans, oscillations underlying aversive learning are not well characterised, largely due to the technical difficulty of recording from these two structures. Here, we used high‐precision magnetoencephalography (MEG) during human discriminant delay threat conditioning. We constructed generative anatomical models relating neural activity with recorded magnetic fields at the single‐participant level, including the neocortex with or without the possibility of sources originating in the hippocampal and amygdalar structures. Models including neural activity in amygdala and hippocampus explained MEG data during threat conditioning better than exclusively neocortical models. We found that in both amygdala and hippocampus, theta oscillations during anticipation of an aversive event had lower power compared to safety, both during retrieval and extinction of aversive memories. At the same time, theta synchronisation between hippocampus and amygdala increased over repeated retrieval of aversive predictions, but not during safety. Our results suggest that high‐precision MEG is sensitive to neural activity of the human amygdala and hippocampus during threat conditioning and shed light on the oscillation‐mediated mechanisms underpinning retrieval and extinction of fear memories in humans.

## 1 INTRODUCTION

One core function of the amygdala is to store associations between neutral and aversive events in a process often termed ‘threat conditioning’ or ‘fear conditioning’, as demonstrated by a body of rodent literature (LaBar & LeDoux, 1996; LeDoux, 2000; Likhtik & Paz, 2015; Maren & Quirk, 2004; Stujenske, Likhtik, Topiwala, & Gordon, 2014). A distributed network of cortical and subcortical brain regions contributes to the creation and recall of this type of memory, including hippocampus, sensory, and prefrontal cortical areas (Herry & Johansen, 2014; Likhtik & Paz, 2015; Stujenske et al., 2014). These latter areas are directly connected to subnuclei of the amygdala (Abivardi & Bach, 2017; Felix‐Ortiz et al., 2013; Janak & Tye, 2015; Lithari, Moratti, & Weisz, 2016; Sah, Faber, Armentia, & Power, 2003) and communicate with amygdala through synchronised firing of neuronal populations (Fries, 2015), which in the case of threat conditioning occurs mainly in the theta range (Adhikari, Topiwala, & Gordon, 2010; Likhtik & Paz, 2015; Paz, Bauer, & Paré, 2008; Seidenbecher, Laxmi, Stork, & Pape, 2003; Stujenske et al., 2014). In particular, theta oscillations are increased in the rodent amygdala and hippocampus during anticipation of threat compared to safety (Seidenbecher et al., 2003; Stujenske et al., 2014) and decreased during states of relative safety, compared to threat (Lesting et al., 2013; Likhtik & Paz, 2015; Stujenske et al., 2014).

Investigating these oscillations in humans has remained a challenge until today, despite advances in neuroimaging technology. While functional magnetic resonance imaging signal is influenced by coordinated neural oscillations (Boorman et al., 2015; Hutchison, Hashemi, Gati, Menon, & Everling, 2015; Scheeringa, Koopmans, van Mourik, Jensen, & Norris, 2016), it does not allow any direct inference on the functionally relevant time–frequency structure (Lisman & Jensen, 2013). On the other hand, noninvasive electrophysiological methods such as magnetoencephalography or electroencephalography (MEG/EEG), have found it difficult to isolate signals from amygdala, due to its small size and deep location. Indeed, most previous threat conditioning studies using MEG did not analyse or report the temporal dynamics of signals specifically emanating from the amygdala (Chien et al., 2017; Kluge et al., 2011; Moses, Bardouille, Brown, Ross, & McIntosh, 2010; Moses, Martin, Houck, Ilmoniemi, & Tesche, 2005; Rehbein et al., 2014; Tesche et al., 2007), while a few reported evoked responses in amygdala (Balderston, Schultz, Baillet, & Helmstetter, 2014; Moses et al., 2007), or indirect evidence of amygdalar contributions to a wider network underlying threat conditioning (Lithari et al., 2016; Lithari, Moratti, & Weisz, 2015). Thus, to date, there is no direct characterisation of amygdala oscillations during human threat conditioning. Nevertheless, simulation studies have demonstrated the feasibility of reconstructing oscillatory activity originating from the amygdala with an appropriate head model (Attal, Maess, Friederici, & David, 2012; Dumas, Attal, Dubal, Jouvent, & George, 2011). Here, we sought to provide a proof‐of‐principle of this possibility, by demonstrating that neural oscillatory signals during threat conditioning and extinction can be detected with high‐precision MEG, and making plausible that the detected signal does indeed emanate from the amygdala rather than other, potentially confounding, sources.

The amygdala, despite its small size relative to surrounding structures, has a neuronal density up to five to six times higher than neocortex (Dumas et al., 2013; Pakkenberg & Gundersen, 1997; Schumann & Amaral, 2005). Thus, an activated volume as small as 0.2–0.3 cm^3^ could suffice to generate a measurable MEG signal (Dumas et al., 2011, 2013), a volume that corresponds to the size of amygdala nucleus groups in humans (Abivardi & Bach, 2017; Bach, Weiskopf, & Dolan, 2011), underlining the feasibility of amygdala source reconstruction. Similarly, the hippocampus has a relatively high source density, at least two times higher than that of the neocortex (Meyer, Rossiter, et al., 2017; Murakami & Okada, 2006). Simulation studies are providing converging evidence on the feasibility of recording hippocampal activity with MEG (Attal & Schwartz, 2013; Mills, Lalancette, Moses, Taylor, & Quraan, 2012; Quraan, Moses, Hung, Mills, & Taylor, 2011; Stephen, Ranken, Aine, Weisend, & Shih, 2005) in particular under a sufficiently low signal‐to‐noise ratio (SNR) and coregistration error (Meyer, Rossiter, et al., 2017), or when the source signal is particularly pronounced, such as for epileptic discharges (Pizzo et al., 2019). Nevertheless, the majority of the available evidence regarding reconstruction of hippocampus/amygdala sources remains theoretical, and/or simulation‐based, where ground truth is known.

The ill‐posed nature of the source reconstruction problem indeed makes it difficult to corroborate that the detected sources correspond to actual neural sources (Lopez, Litvak, Espinosa, Friston, & Barnes, 2014). This is because different solutions to the problem—that is, different reconstructed sources—could have generated the same, or a very similar, sensor‐level signal. Source‐reconstruction techniques typically use constraints on the prior distribution of sources and on the structure of noise to find a unique solution (Lopez et al., 2014), such that different constraints can lead to different solutions. Here, we address this problem by varying the anatomical forward model—the region in space where sources are allowed. By completely removing, or shifting in space, the location of the amygdala or hippocampus, we analyse whether the best solution under this altered forward model explains the sensor‐level data as well as with the amygdala/hippocampus location included. To avoid that a specific set of reconstruction constraints is responsible for our findings, we repeat this analysis with two common source reconstruction algorithms and under different implementations.

To achieve an optimal SNR for this approach, we minimised two major sources of imprecision, head movement, and coregistration errors, with a recently introduced high‐precision MEG technique, where participant‐specific flexible headcasts stabilise their head inside the MEG system and limit head movement within, and between, sessions (Meyer, Bonaiuto, et al., 2017; Troebinger, López, Lutti, Bradbury, et al., 2014). Simulation studies have demonstrated the potential for headcast‐based MEG recordings to discriminate cortical layers (Bonaiuto, Meyer, et al., 2018; Troebinger, Lopez, Lutti, Bestmann, et al., 2014) and reconstruct hippocampal neural activity (Meyer, Bonaiuto, et al., 2017), but there is no experimental evidence yet for the feasibility of this technique to localise oscillations from subcortical structures. Furthermore, instead of scanning a large number of participants, we sought to minimise noise on the within‐participant level, by choosing a large number of trials, which is a standard approach in nonhuman primate electrophysiology (Klavir, Genud‐Gabai, & Paz, 2013) and precision functional mapping (Gordon et al., 2017). Specifically, we tested the hypothesis that theta and gamma oscillations in human amygdala and hippocampus during maintenance and extinction of aversive memories can be detected with high‐precision MEG. Moreover, we performed exploratory analyses on how theta and gamma oscillations originating from the hippocampus and amygdala are modulated by states of threat versus safety in humans.

## 2 MATERIALS AND METHODS

### 2.1 Participants

We recruited five volunteers (two females), aged 21 to 31 years old (mean age ± *SD*: 27 ± 5 years old). All volunteers reported no history of neurological or psychiatric disorder and normal or corrected to normal vision and hearing. Participants gave their written informed consent prior to their participation. The study, including the form of taking consent, was approved by the University College London (UCL) research ethics committee (project ID: 6649/001).

### 2.2 Experimental procedure

**Figure 1:**
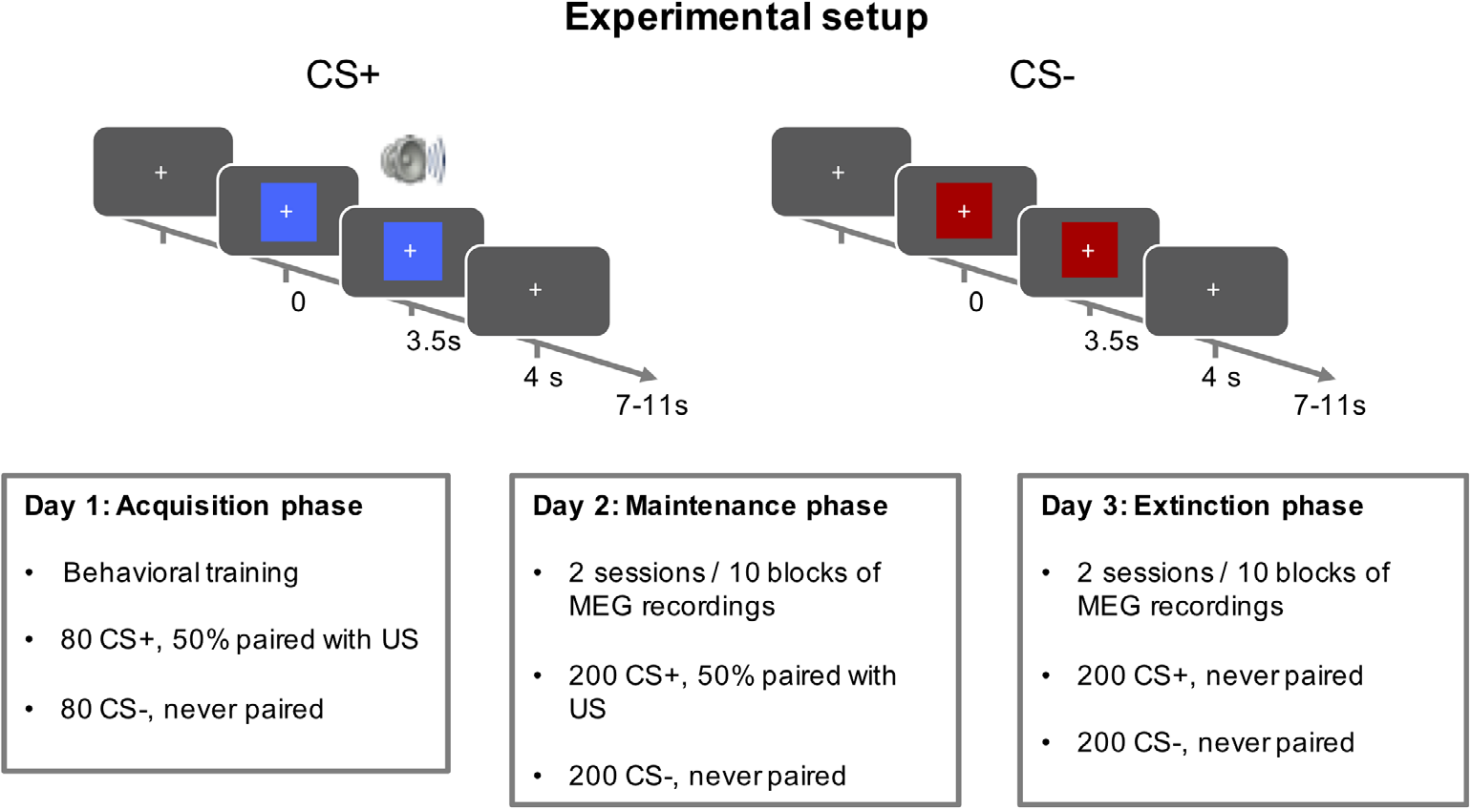
Experimental setup. The experiment consisted of three phases, on three consecutive days. In Phase 0, participants were trained on the CS/US association outside MEG. Phase 1 (maintenance) and Phase 2 (extinction) took place inside an MEG scanner, and were split into 10 sessions each. CS, conditioned stimuli; MEG, magnetoencephalography; US, unconditioned stimulus [Color figure can be viewed at http://wileyonlinelibrary.com]

The experiment consisted of five sessions, conducted on three consecutive days (Figure 1). Session 0 was an acquisition session, during which participants were exposed to the conditioned stimuli/unconditioned stimulus (CS/US) association outside the MEG scanner. Sessions 1–4 (maintenance and extinction) took place in the MEG scanner.

In each session, participants were presented with one of two different CSs for 4 s, which were a red and blue rectangle. In acquisition and maintenance phases, one of the two CS (CS+) coterminated on 50% of the trials with an US, while the other CS (CS−) was always presented without a US. In the extinction phase, no US was presented. US were 500 ms, 95 dB white noise bursts. We used noise bursts rather than other commonly used US (i.e., mild electric shocks), as they have been suggested to elicit more stable conditioned responses in experiments involving repetitions over a large number of trials (Sperl, Panitz, Hermann, & Mueller, 2016). US started 3.5 s after CS onset and coterminated with the CS. They were presented via in‐ear pneumatic headphones during all MEG sessions, and with overhead headphones during the acquisition (non‐MEG) phase.

Trials were presented in a random order with a 7–11 s intertrial interval. In the maintenance and extinction phases, participants were presented with blocks of 40 trials, lasting approximately 10 min each. In acquisition Session 0, preceding MEG, participants were presented with two blocks of 80 trials each. This resulted in 160 acquisition trials, 400 maintenance trials, and 400 extinction trials. Participants were instructed to attend to the visual stimuli and indicate, via button press, the colour of the stimulus that was presented to them on each trial, irrespective of whether a US was present or not. Association between CS and colour/key was randomised over participants, but was kept unchanged within sessions of each individual participant.

### 2.3 Extraction of anatomical information

Prior to MEG sessions, participants underwent two Magnetic Resonance Imaging (MRI) scanning protocols during the same visit: one to generate the scalp image for production of the headcast, and a second one used for MEG source localization. Structural MRI data for all participants were acquired on a Siemens Tim Trio 3T system (Erlangen, Germany) in supine position, as described previously (Meyer, Bonaiuto, et al., 2017).

The first protocol was used to generate an accurate image of the scalp for headcast construction (Meyer, Bonaiuto, et al., 2017). A radiofrequency (RF) and gradient spoiled T1 weighted three‐dimensional (3D) fast low angle shot (FLASH) sequence was used, with 1 mm^3^ resolution (1 mm slice thickness), field‐of view 256, 256, and 192 mm along the phase (A–P), read (H–F), and partition directions, as in previous high‐precision MEG studies (Bonaiuto, Meyer, 2018; Meyer, Bonaiuto, et al., 2017). For this protocol, data were acquired with a 12‐channel head coil. Acquisition time was 3 min 42 s, plus a 45 s localizer sequence.

The second protocol was a quantitative multiple parameter mapping (MPM) protocol, and it was used to extract cortical, amygdalar, and hippocampal surfaces. This consisted of three differentially weighted, RF, and gradient spoiled, multiecho 3D FLASH acquisitions, with whole‐brain coverage at 800 μm isotropic resolution, as described previously (Bonaiuto, Meyer, 2018). For this protocol, data were acquired with a 32‐channel head coil to increase SNR. A T1 image was also acquired from the MPM protocol, with the 32‐channel coil and it was then used to align the MPM volumes to the 1 mm isotropic scan. The flip angle for the T1 weighted acquisition was 21°, and the field of view was set to 224, 256, and 179 mm along the phase (A–P), read (H–F), and partition (R–L) directions, respectively (Bonaiuto, Meyer, et al., 2018). Total acquisition time for all MRI scans was less than 30 min.

Quantitative maps of proton density (PD), longitudinal relaxation rate (R1 = 1/T1), magnetization transfer saturation, and effective transverse relaxation rate (R2* = 1/T2*) were subsequently calculated according the procedure described in Weiskopf et al. (2013). All quantitative maps were coregistered to the scan used to design the headcast, using the T1 weighted map, and were then used to extract cortical surface meshes using FreeSurfer (see below).

#### 2.3.1 Headcast construction

For headcast construction, scalp surfaces were extracted from the first T1‐weighted image using SPM12 (http://www.fil.ion.ucl.ac.uk/spm/). These were then converted to a standard template library format, which is suitable for 3D printing, and were combined with a 3D model of the fiducial coils as described previously (Meyer, Bonaiuto, et al., 2017). This 3D model was placed inside a virtual version of the scanner dewar helmet, by minimising the sensor‐to‐head distance. The resulting head model was 3D printed and placed inside a replica of the dewar helmet. The headcast was then constructed by pouring liquid resin between the 3D head model and dewar‐helmet replica.

#### 2.3.2 Extraction of cortical and hippocampal surfaces

To extract cortical and hippocampal surfaces from the MRI data of all participants, we used FreeSurfer v5.3.0 (Fischl, 2012) as described previously (Bonaiuto, Meyer, et al., 2018; Bonaiuto, Rossiter, et al., 2018; Meyer, Rossiter, et al., 2017; Troebinger, Lopez, Lutti, Bestmann, et al., 2014). In particular, an in‐house FreeSurfer surface reconstruction procedure (Bonaiuto, Meyer, et al., 2018), using the PD and T1 maps as inputs, was used to extract cortical and hippocampal surfaces for the special case of multiparameter maps, as these might lead to localised tissue segmentation failures under standard procedures, because the boundaries between the pial surface, dura mater, and Cerebrospinal fluid (CSF) show different contrasts in these images with respect to that assumed within FreeSurfer (Lutti, Dick, Sereno, & Weiskopf, 2014). Hippocampal surfaces were generated by extracting isosurfaces, using the isosurface function in MATLAB (version 2015A; MathWorks, Natick, MA), from the subcortical segmentation created by FreeSurfer v5.3.0. downsampling the resulting meshes by a factor of 10 to match the cortical surface.

#### 2.3.3 Extraction of amygdalar surfaces

To account for its relative small size, amygdala boundaries were traced manually as opposed to coarser automated methods. Manual segmentation of the participants' amygdalae was implemented in FSLView (Jenkinson, Beckmann, Behrens, Woolrich, & Smith, 2012) by comparing individual T1‐weighted images with schematic illustrations of the anatomical atlas by Mai, Majtanik, and Paxinos (2015), following the protocol described in Bach, Behrens, et al. (2011) and Abivardi and Bach (2017). Crucially, we used the hippocampus, optical tract, temporal horn of the lateral ventricle, and sulcus semiannularis as guiding landmarks to delineate outer amygdala borders, advancing from posterior to anterior in coronal slices. The resulting masks were subsequently corrected in sagittal, axial, and one more time in coronal orientation. Particular care was taken not to include any hippocampal voxels in the segmentation. In a final step, amygdala masks were automatically smoothed with the SPM functions spm_erode and spm_dilate (SPM12, [http://www.fil.ion.ucl.ac.uk/spm/software/spm/]). Both functions use a standard 3 × 3 × 3 binary voxel kernel. Smoothing was applied to minimise irregular 3D mask shape arising from the manual segmentation procedure using three planes (axial/coronal/sagittal), as in our previous work (Abivardi & Bach, 2017; Bach, Behrens, et al., 2011).

The mean volume of the extracted hippocampal volumes across participants was 3,819 ± 154 mm^3^ and 3,900 ± 255 mm^3^ for the left and right hippocampi, respectively. For the amygdalae, the mean volume across participants was 975 ± 42.3 mm^3^ and 980 ± 40.7 mm^3^ for the left and right amygdalae, matching closely previous results on a different group of participants, using the same technique (Abivardi & Bach, 2017). We subsequently approximated the surface of the hippocampi and amygdalae with triangular meshes. The vertex‐to‐vertex distance of the extracted meshes, which is indicative of the mesh resolution, was matched across participants/regions and was on average 4.52 ± 0.06 mm for cortical meshes, 4.42 ± 0.09 mm for amygdala and 3.45 ± 0.01 mm for hippocampus. In total, these meshes contained an average number of 8,196; 57; and 480 vertices, for the cortex, amygdala, and hippocampus, respectively.

### 2.4 Acquisition of MEG data

All MEG recordings were performed in a magnetically shielded room, using a 275‐channel Canadian Thin Films system with superconducting quantum interface device‐based axial gradiometers. Data were acquired with a hardware anti‐alias filter of 150 Hz cutoff frequency and sampling rate of 600 Hz. Prior to entering the scanners, participant‐specific flexible headcasts were placed on participants heads and head positioning coils were attached inside the headcasts, at the nasion and left and right auricular sites, using indentations in the headcast with known MRI‐space coordinates. Positioning coils were used for continuous head localization and anatomical coregistration. CS was displayed through a projector on a screen, positioned at 0.8 m from the participants' eyes. US was presented to participants through in‐ear pneumatic headphones.

### 2.5 Preprocessing of MEG data

We used SPM 12 (Statistical Parametric Mapping; Wellcome Centre for Human Neuroimaging, London, UK; http://www.fil.ion.ucl.ac.uk/spm) for analysis of MEG data. Continuous data from each session were notch filtered around 50 Hz and downsampled to 300 Hz. Peri‐stimulus epochs were extracted from −1 to 4 s poststimulus onset. Eyeblinks were detected automatically on frontal MEG sensors (‘MZF01’) and their onset was marked in the MEG files. This relied on eyeblink artefact rejection utilities implemented in SPM, by filtering the data at 1–15 Hz, detecting outliers in single‐trial data and marking outliers that exceeded 4 standard deviations of the trial‐by‐trial data distribution. Eyeblinks were then visually inspected to ensure their successful detection. As our analyses did not change qualitatively by excluding trials with detected eyeblinks, we kept all trials for subsequent analyses. Moreover, the relative location of three fiducial coils (LPA, RPA, and nasion) during each session was extracted to estimate head movement for all sessions/participants (Supporting Information 1).

### 2.6 Source localisation

To increase precision in source localization, we constructed anatomical models for each individual participant, by combining the meshes that were obtained by segmenting their MRI scans. In particular, for each participant, we constructed four anatomical models: the first only contained a cortical mesh and the remaining models a cortical together with either amygdala or hippocampus mesh, or all three meshes combined.

To reconstruct neural activity, we used a multiple sparse priors (MSP) algorithm (Friston et al., 2008), which strives to maximise the explained variance in the measured data using the minimal number of sources. MSP has been previously shown to recover simulated data from deep sources originating in the hippocampus with higher likelihood compared to other inversion schemes, such as beamformers and minimum norm estimates (Meyer, Rossiter, et al., 2017). We additionally employed an Empirical Bayesian Beamformer (EBB) (Belardinelli, Ortiz, Barnes, Noppeney, & Preissl, 2012), which effectively weights an empirical prior distribution of sources—assuming only local covariance—against a uniform sensor noise distribution and uses the covariance at sensor level to form a prior of the covariance at source level (Supporting Information 2). In total, we performed four model inversions per participant and algorithm, one for each head model.

For both algorithms, we computed source localization using all single trials, from both experimental conditions (CS+/−) of the maintenance phase, across the whole anticipatory interval from CS onset, up to US onset or omission (i.e., 0–3,500 ms post‐CS onset) and used a broadband frequency range of 1–120 Hz. We used the maintenance phase for model selection, as we expected to have stronger responses in the hippocampus and amygdala during this phase, compared to the extinction. We additionally report model selection results for the extinction phase (Supporting Information 6 and Supplemental Figure 6). Before inversion, MEG data are decomposed through singular value decompositions by reducing them to a number of orthogonal channels (spatial modes) and time points (temporal modes). We kept the number of spatial and temporal modes constant, and equal to the default values (*N* = 120 and 4, respectively). In the inversions, each single trial contributed to a running average of the sensor level covariance matrix, which was then approximated (through the Bayesian scheme) with an expanded source level covariance matrix (based on a mixture of MSP components) plus a sensor level noise term.

**Figure 2:**
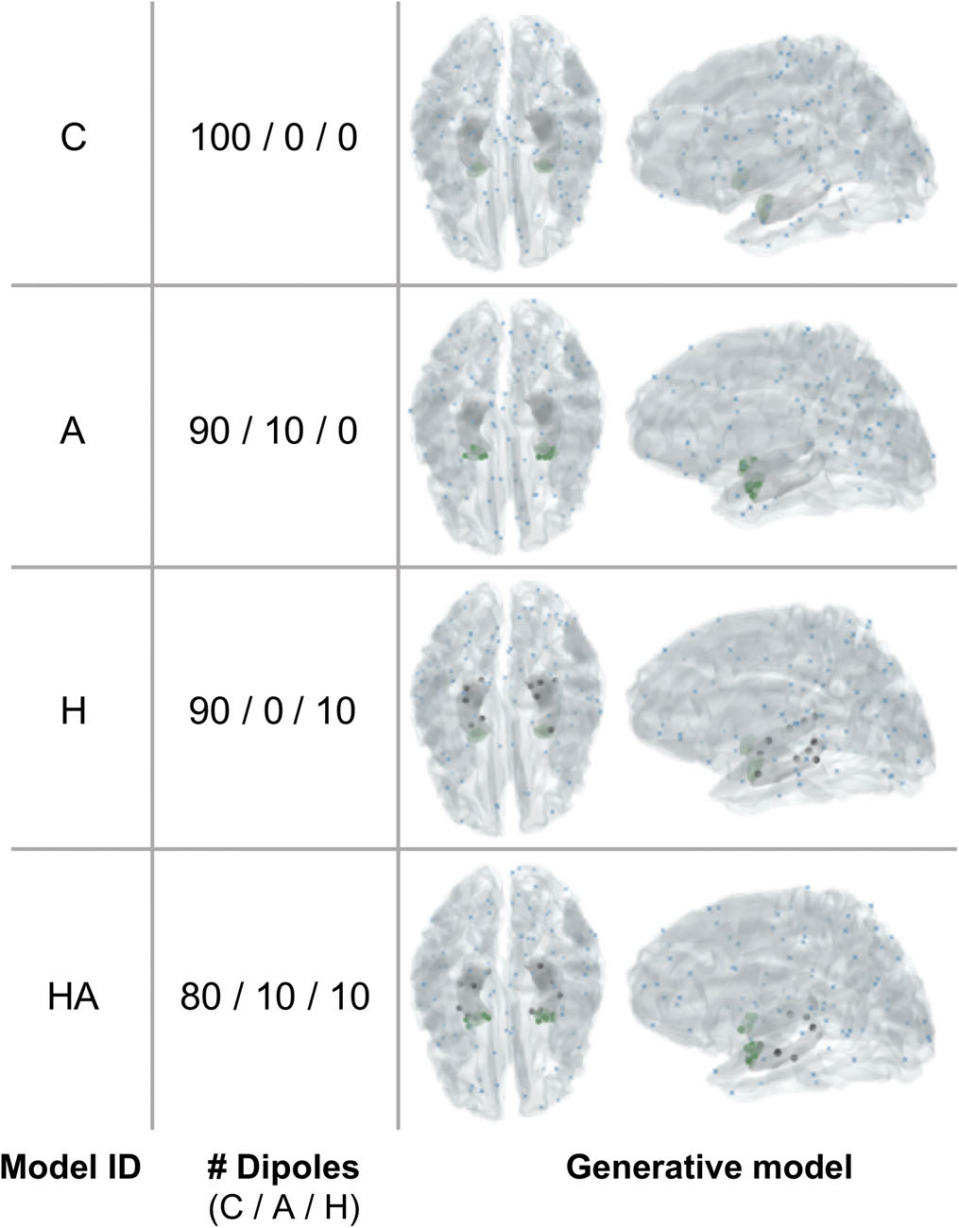
Distribution of sparse sources for one exemplar participant, obtained with four generative models, after multiple sparse priors optimization. All models contain 100 sources, distributed either on the cortex (C) only (blue dots), or on the cortex and hippocampus (H) (grey dots), and/or amygdala (A) (green dots) [Color figure can be viewed at http://wileyonlinelibrary.com]

For all MSP inversions, we used the same head model, including meshes for the cortex, hippocampus, and amygdala, but we changed the way that the source priors are distributed across these meshes. As we were interested in localising very sparse sources, we only included 100 priors for all inversions and these were distributed pseudorandomly, such that the exact location of priors on a given mesh was always randomly set, but their proportion on each mesh (i.e., cortical, hippocampal, amygdalar), was fixed. The exact total number of priors in our models is not informative of the underlying neural processes and thus is of little interest for the present analysis; what is important is that we kept the number of priors the same for all examined models, so that all models have the same complexity. Indeed, inclusion of a higher number of priors (*N* = 500 priors), did not change the pattern of results (Supporting Information 5 and Supplemental Figure 5). Across different models, priors were placed either all on random locations on the cortex (for Model C), or were split among random locations on the cortex and the bilateral hippocampal (Model H) (10%) and amygdala (Model A) (10%) meshes, or over all meshes (80% on the cortex, 10% in the amygdala, and 10% in the hippocampus—model HA) (see Figure 2 for an overview of all models and patch locations for one exemplar participant). To ensure convergence of the MSP algorithm, we implemented an iterative version, by initiating the set of random patches 16 times and retaining the one that maximised the Free Energy across iterations (Lopez et al., 2014).

For the EBB algorithm, we used its standard implementation in SPM12. For each inversion, we estimated as many sources as the total number of vertices that were included in the head model. These ranged between 8,196, for the standard cortical only mesh, plus the additional vertices that would result from amygdala and hippocampal meshes, if present. The subcortical meshes were nested inside the cortical mesh, and for both EBB and MSP inversions, the meshes constrain the source locations and orientations to be at the mesh vertices and normal to the mesh surface (see Section 4 for further details on how cell morphology and organisation relates to the models).

### 2.7 Model selection

In order to select the model that is more likely, given the recorded MEG data, we approximated model evidence by the negative free energy (F) as a variational Bayes approximation to model evidence (Friston, Mattout, Trujillo‐Barreto, Ashburner, & Penny, 2007; Lopez et al., 2014; Meyer, Rossiter, et al., 2017; Troebinger, Lopez, Lutti, Bestmann, et al., 2014). Like other approximations to model evidence, free energy provides a tradeoff between model accuracy and model complexity (accuracy is rewarded, complexity is penalised to minimise overfitting). F is on the scale of log‐likelihood, and higher values reflect higher model evidence or ability to fit the data with more parsimonious models. We considered an F difference between the combined model (cortex + amygdala and/or hippocampus) and cortical model of 3 (or ~20 times more likely) or more as decisive, in line with Bayesian conventions (Penny, Stephan, Mechelli, & Friston, 2004).

### 2.8 Time–frequency analysis

Having established a winning model based on the task period data exclusively, we re‐estimated the source reconstruction for this model, but by extending the extracted window to cover also 1 s of baseline interval, to minimise the influence of potential edge effects. We extracted the time courses corresponding to the source (per region and participant) with the maximum estimated current, on average, across all experimental conditions. We repeated all analyses by considering the average time course of activity per region across all sources, and results were qualitatively similar.

For each reconstructed time course, we computed a time–frequency decomposition using Morlet wavelets, in a temporal window of −1,000 to 3,500 ms poststimulus onset with 3 ms resolution, and a frequency window of 1–120 Hz, with 1 Hz resolution, similar as in a previous study using MEG to reconstruct hippocampal oscillations (Khemka, Barnes, Dolan, & Bach, 2017). The frequency for the wavelets was derived from previous studies using similar paradigms in rodents (Stujenske et al., 2014), while taking into account the equivalent of rodent theta in humans (Jacobs, 2014).

We performed both exploratory and hypothesis‐driven analyses on the time–frequency decomposed signals, at group and single‐participant level (Supporting Information 3). For the former, we contrasted oscillatory power between CS+ and CS− trials, for each time and frequency point, seeking differences that were time locked to the CS occurrence. For the latter, we focused on three predefined frequency bands, derived from previous studies of threat conditioning in rodents (Stujenske et al., 2014) and their homologous bands in humans (Jacobs, 2014). These were theta (1–8 Hz), low gamma (30–70 Hz), and high gamma (70–120 Hz). For this part of the analysis, we considered the mean power over the whole poststimulus interval (0–3,500 ms), including evoked and induced oscillatory activity.

### 2.9 Phase locking analysis

We assessed patterns of neural synchrony between the hippocampus and amygdala using the phase lag index (PLI) (Stam, Nolte, & Daffertshofer, 2007), which quantifies consistent phase differences between two signals of interest. Compared to other metrics of neural synchrony, the PLI is less prone to volume conduction confounds from common sources (Kaplan et al., 2012; Stam et al., 2007) and has been previously used as an index of neural synchrony between the hippocampus and other brain areas, using MEG (Guitart‐Masip et al., 2013; Khemka et al., 2017).

We filtered the time series extracted from the hippocampus and amygdala in the theta frequency band (1–8 Hz) using a fourth‐order bidirectional Butterworth filter. The instantaneous phase φ_*i*_(*t*, *n*) of the filtered time series for each region (*n*), time point (*t*), and trial (*i*) was derived using the Hilbert transform. The PLI was calculated using the formula:

$${\mathrm{PLI}}_{i}=\frac{1}{T}\left|\sum_{t=1}^{T}\mathrm{sign}\left[\varphi_{i}\left(t,H\right)-\varphi_{i}\left(t,A\right)\right]\right|$$

where *H* and *A* denote time series derived from the hippocampus and amygdala, respectively. PLI ranges from 0, for no coupling, to 1, for a constant phase difference across the two time courses of interest, which is indicative of synchrony.

### 2.10 Statistical analysis

We first performed exploratory analyses in all time points and frequency bands, to identify potential time‐locked effects of threat conditioning. To this end, we computed post hoc *t* tests between CS+ and CS− trials, on the logarithm of the power for each time–frequency bin. Correction for multiple comparisons across time and frequency was performed at the cluster level, using a random permutation test on the trial labels with 1,000 iterations (Maris & Oostenveld, 2007).

Additionally, we contrasted differences in power in a priori defined frequency bands on the single‐trial level, using linear mixed effect (LME) models (package nlme) in R (http://www.r-project.org; version 3.2). Given our experimental design, we considered three fixed effects factors: CS (CS±), region (amygdala/hippocampus), and time (number of blocks). The random effect structure was established using model selection (Westfall, Nichols, & Yarkoni, 2017) (Supporting Information 4 for more details). Specifically, we included into our model, a random intercept and a random region factor over participants, to account for interparticipant variance. We used the R model formula:

$$\text{Power}<-1+\mathrm{CS}\times \text{Time}\times \text{Region},\text{random}=∼\left(1+\text{Region}\right)|\text{Participant}$$

The same model but without the factor ‘Region’ was used to test effects of CS and time on the PLI:

PLI<−1+CS×Time,random=∼1∣Participant

When plotting results of LME models (Figures 6b and 7), we additionally display a linear fit of the mean responses over time, derived with R function ‘stat_smooth()’, with method = ‘lm’. Results from this analysis were Bonferroni corrected to account for the multiple comparisons across frequency bands.

## 3 RESULTS

### 3.1 Model comparison

We investigated whether a generative biophysical model allowing sources in hippocampus and/or amygdala described the MEG data better than a model that permitted cortical sources only. This spatial selectivity was encoded in Bayesian priors (Mattout, Phillips, Penny, Rugg, & Friston, 2006). We reasoned that if the deeper sources are indeed contributing to the measured signal, then including them in our generative model should better describe the data at the sensor level.

**Figure 3:**
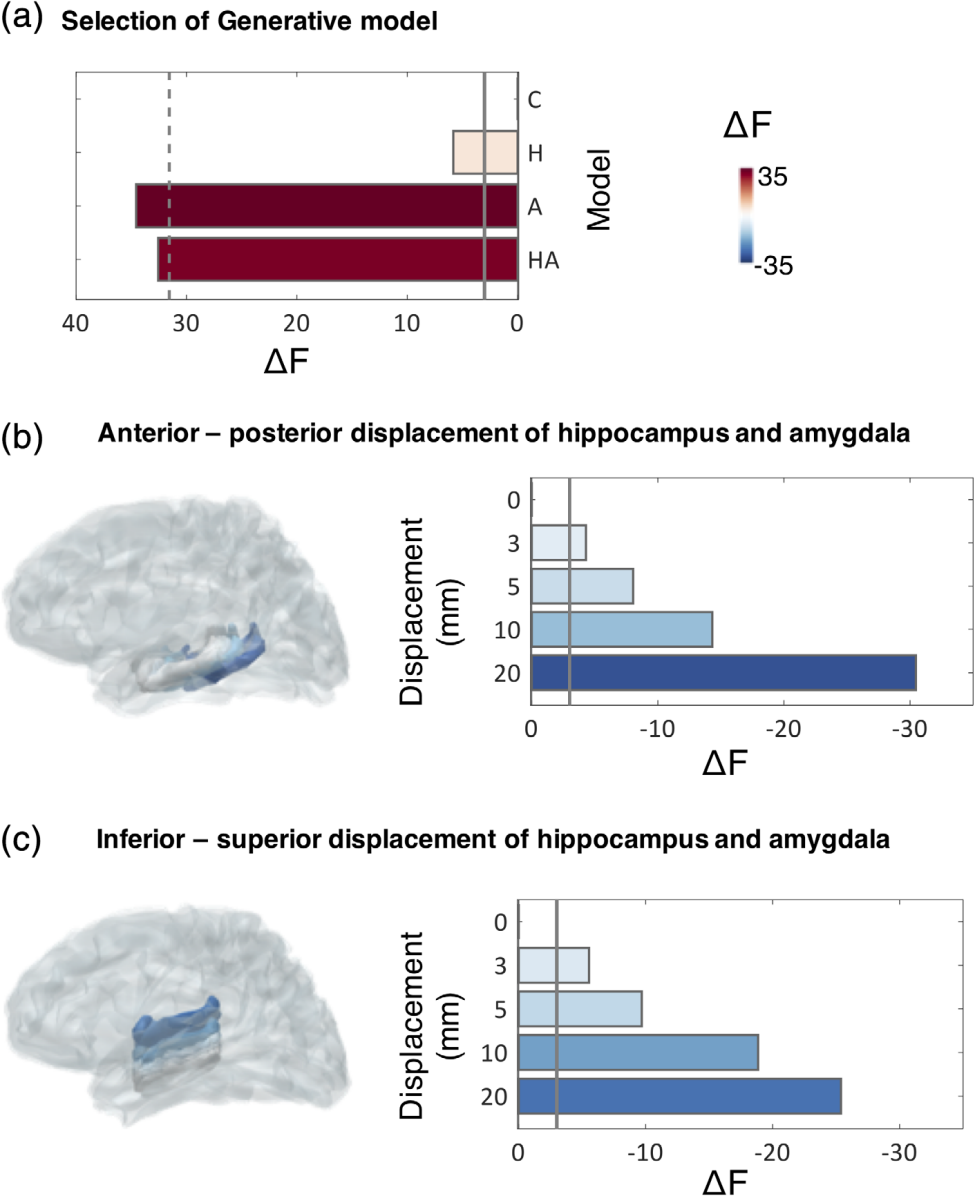
Comparison of generative models. (a) Free energy (*F*) increases by including sparse priors on either the hippocampal (H) or amygdalar (A) surface meshes, or both (HA), compared to a baseline model containing only cortical sources (C). The solid line displays the threshold for a significant increase in *F* values with respect to Model C (i.e., Δ*F* = *F*–*F*_C_), while the dashed line denotes a significant decrease with respect to the model with the highest *F* value (Model A, i.e., *F*_A_‐3). For Models A and HA, there is a significant increase in model evidence, compared to baseline Model C (*F*_A_–*F*_C_ = 34.56, *F*_HA_–*F*_C_ = 32.56). All *F* values are summed across participants, as is typically done in a fixed effects design. (b,c) Change in *F* values as a result of displacing the hippocampus and amygdala meshes along the anterior–posterior (B), or inferior–superior (C) axes (depicted to the left of each plot). A displacement of 3 mm is sufficient to result in a significant decrease in *F*. This is defined as a decrease by more than three log units with respect to the original model (solid line). The plots to the left illustrate the displaced meshes, colour coded according to the change of *F* values across participants. Grey meshes depict the original MRI‐derived locations [Color figure can be viewed at http://wileyonlinelibrary.com]

We used two different algorithms for model inversion, MSP, (Friston et al., 2008), an algorithm which combines and prunes possible priors in order to maximise the evidence of a model in explaining the measured data and EBB (Belardinelli et al., 2012), which generates an empirical prior source level distribution based on the assumption that sources are predominantly locally covariant. We compared four different generative models per participant (cortex only [Model C], cortex and hippocampus [Model H], cortex and amygdala [Model A], and cortex and both amygdala and hippocampus [Model HA]) (Section 2.6). We found that using MSP, MEG data were best explained under Model A, followed by model HA (Figure 3a). The evidence for these two best models was not significantly different (*F*_A_–*F*_HA_ = 2.00), but for both models, evidence was significantly higher than for the baseline Model C, in accordance with our hypothesis (*F*_A_–*F*_C_ = 34.56, *F*_HA_–*F*_C_ = 32.56). This suggests that sensor‐level MEG data, at 1–120 Hz were significantly better explained by a generative model including sources in both hippocampus and amygdala, compared to a model with no deep sources, in turn suggesting that an apparent contribution of deep sources cannot be explained by confounding cortical sources alone. These results were largely replicated with a less sparse implementation of MSP including 500 priors (Supporting Information 5), and with a different inversion algorithm, EBB (Supporting Information 2). Moreover, model selection at the group level was in line with model selection results obtained for single participants, which showed that adding priors in the hippocampus and/or amygdala resulted in a decisively better model for all but one participants using MSP, and for all participants with EBB (see Supporting Information 3 for single‐participant analyses).

Next, we evaluated spatial sensitivity to underlying brain anatomy, assessing the precision in localisation of deep sources. To assess this, we displaced the bilateral hippocampal and amygdala meshes along the anterior–posterior and inferior–superior axes (into one direction at a time to avoid displacements outside the brain) and repeated the source reconstruction while keeping the same initial locations for the sources relative to the mesh (which thus move spatially when the meshes are moved). Results from this analysis showed that model evidence decreased markedly when the sources used to explain the data were moved in space (Figure 3b,c). A displacement of 3 mm of the hippocampal/amygdalar mesh was sufficient to result in a significant decrease in model evidence (Figure 3b,c) which reflects the spatial sensitivity of our approach. This also suggests that the HA model is not simply explaining unrelated sources of variance from nearby meso‐temporal structures or artefacts. We repeated the same analysis with EBB, which resulted in a similar pattern of results, but a poorer spatial resolution (Supporting Information 2). From here onwards, we therefore report results obtained using MSP and the full model, HA.

### 3.2 Neural oscillations in the hippocampus and amygdala are modulated by potential threat

**Figure 4:**
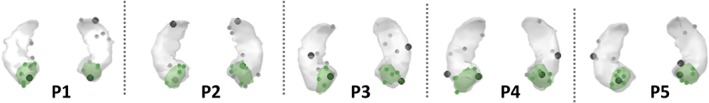
Overview of hippocampus (grey) and amygdala (green) meshes per participant. Small dots highlight the sources retained by multiple sparse priors, while large dots highlight the patches with maximum current per region. The total priors (*N* = 100 priors) in the full model (HA) were pseudorandomly distributed on the cortex, hippocampus, and amygdala (80/10/10 priors in each region, respectively). The retained sources provided a global coverage of the hippocampus and amygdala, and were determined iteratively, by maximising *F* values. Small dots highlight all the retained sources and larger dots the sources with maximal current per region [Color figure can be viewed at http://wileyonlinelibrary.com]

Next, we used model HA obtained with MSP and extracted time courses of source activity in response to CS+ and CS− from sources on the hippocampus and amygdala meshes. In determining the source locations, we collapsed all CS± trials to avoid any circular inference. For each region and hemisphere, we identified the source with the highest amplitude for each structure (*N* = 4 sources per participant: left and right hippocampus and amygdala), and extracted a single time course from each of these sources per participant (Figure 4). We used wavelet transforms to compute time‐frequency decompositions for each individual trial.

**Figure 5:**
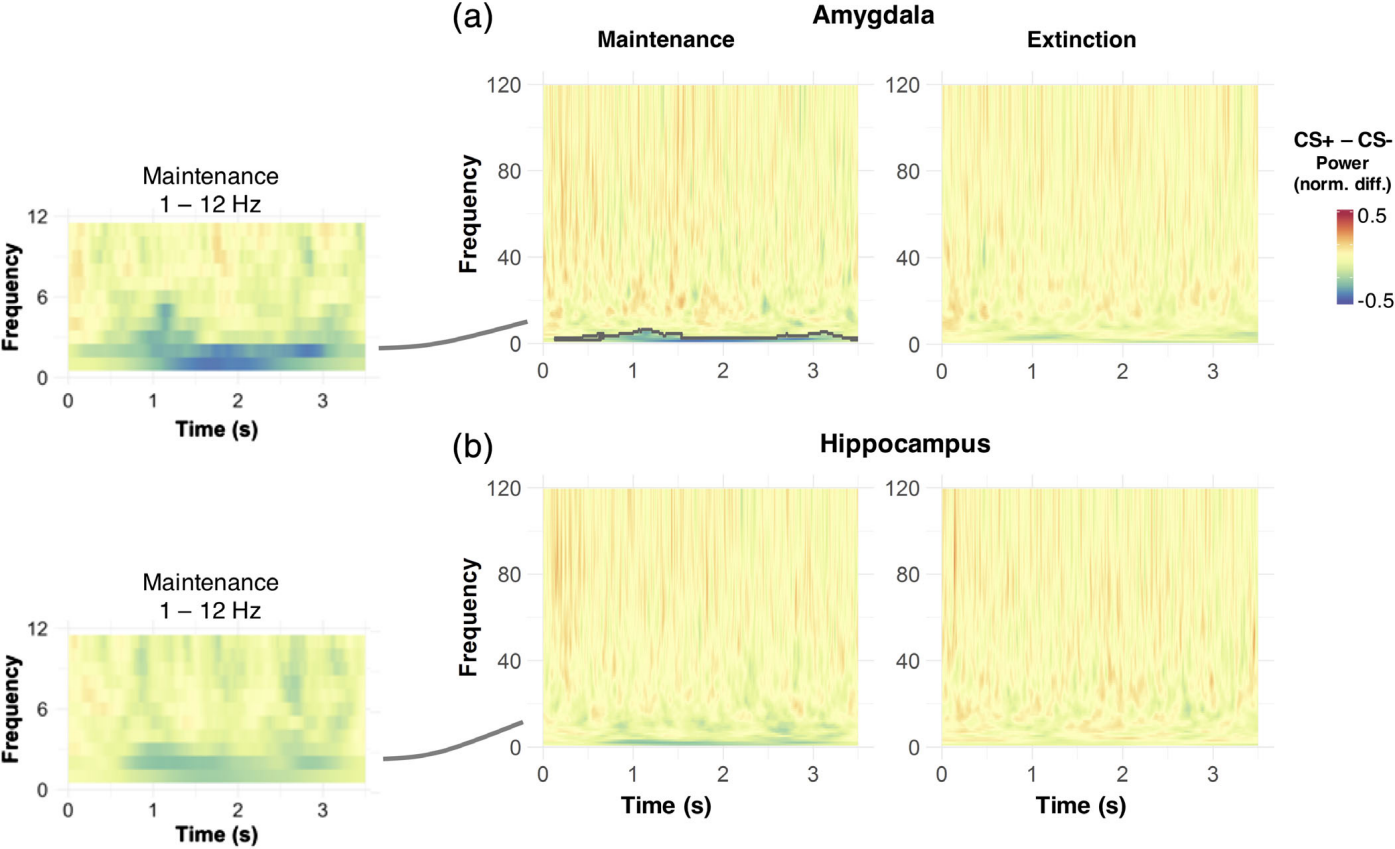
Time–frequency decomposition across participants, for neural responses originating from the hippocampus and amygdala. The contrast between conditioned stimuli (CS+) and CS− stimuli revealed a sustained reduction in oscillatory power of low oscillations, which is mostly pronounced in the amygdala during the maintenance phase and lasts over the whole CS presentation. Grey lines in the full frequency plots to the right highlight time‐frequency clusters with significant differences between CS+ and CS− stimuli (unpaired *t* tests, corrected by random permutations at the cluster level, *p* = .03). To account for interindividual differences in the strength of reconstructed oscillations, all plots are normalised by the maximum single‐trial power value within each participant. Plots to the left illustrate the mean power difference over a smaller frequency range (1–12 Hz) than the ones to the right, for display purposes [Color figure can be viewed at http://wileyonlinelibrary.com]

During the maintenance phase, we observed a significantly lower power in amygdala oscillations during CS+ compared to CS− presentation (Figure 5; corrected at the cluster level with random permutation tests, *p* = .03). This condition difference was particularly pronounced for slow oscillatory power (≤6 Hz) and was evident from around 130 ms poststimulus onset up until trial end (Figure 5). Although a similar decrease in oscillatory power during CS+ was also observed within the hippocampus, this decrease was not significant when correcting for multiple comparisons. During the extinction phase, the power of low frequency oscillations in the amygdala was lower for CS+ compared to CS− trials, but different from the maintenance phase, this decrease was not significant after correction (Figure 5).

### 3.3 Theta and gamma oscillations during maintenance and extinction of fear memories

Based on previous studies in threat conditioning (Stujenske et al., 2014), we had a strong a priori hypothesis that cues predicting safety versus threat would elicit differential neuronal oscillations in the hippocampus and amygdala in specific frequency bands, namely, theta (1–8 Hz in humans) (Jacobs, 2014), low, and high gamma (30–70 and 70–120 Hz, respectively). Consequently, we next focused on these three frequency bands and averaged oscillatory power per trial across the frequency band and the whole poststimulus interval, using an LME model, with fixed effects CS, time, and region. Results were corrected for the number of frequency bands considered.

**Figure 6:**
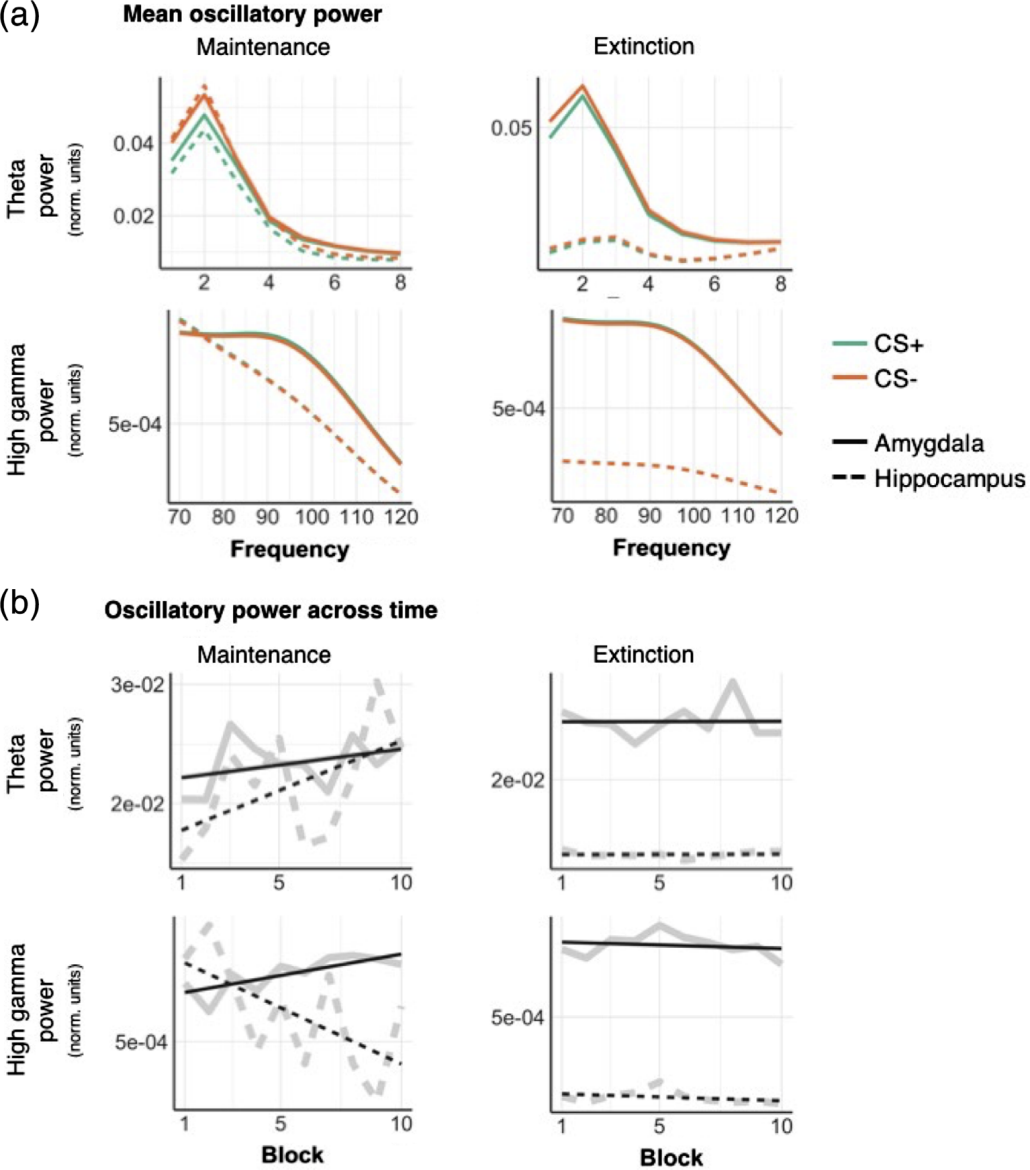
Mean oscillatory power in theta and high gamma frequency bands for CS+ and CS−, reconstructed from sources in the hippocampus (dashed) and amygdala (full lines). (a) Mean theta (1–8 Hz) power was higher for CS−compared to CS+ stimuli, in both the hippocampus and amygdala, during maintenance and extinction phases. In the high gamma band (70–120 Hz), oscillatory power was greater in amygdala than hippocampus, regardless of condition. (b) Mean power for each block, experimental phase and frequency band. All plots are normalised by the maximum single‐trial power value within each participant. Full grey lines represent a linear fit of the power values over time. Full results of the statistical comparison of these plots are displayed in Table 1. Single participant results are displayed in Supporting Information 3 [Color figure can be viewed at http://wileyonlinelibrary.com]

During the maintenance phase, oscillatory power in the theta, but not gamma range, was significantly lower in response to CS+ compared to CS− (Figure 6a, Table 1) in both hippocampus and amygdala, in line with our initial analysis (Figure 5). We found a similar, albeit much weaker, difference for the extinction phase (*F*(1,7984) = 4.68, *p*_uncorr_ = .03, Table 1, Figure 6a), which missed the Bonferroni‐corrected significance threshold of α = .05/3. During maintenance, theta power increased for both regions, with a steeper increase for the hippocampus compared to amygdala, as shown by a time × region interaction (Table 1).

**Table 1:** Statistical analysis of the mean power in theta, low, and high gamma frequency bands. Bold entries are significant (*p* < .05) before correction for multiple comparisons

	Maintenance						Extinction					
	Theta 1–8 Hz		Low gamma 30–70 Hz		High gamma 70–120 Hz		Theta 1–8 Hz		Low gamma 30–70 Hz		High gamma 70–120 Hz	
	*F*(1,7836)	*p*‐Value	*F*(1,7836)	*p*‐Value	*F*(1, 7,836)	*p*‐Value	*F*(1, 7984)	*p*‐Value	*F*(1,7984)	*p*‐Value	*F*(1,7984)	*p*‐Value
**CS**	18.27***	**<.0001**	0.08	.77	0.06	.81	**4.68**	**.03**	0.08	.77	0.23	.63
**Time**	16.28**	**.0001**	94.42***	**<.0001**	13.96**	**.0002**	0.01	.93	2.97	.08	**5.69**	**.02**
**Region**	0.02	.89	0.03	.87	0.05	.82	2.99	.08	2.81	.09	2.80	.09
**CS:Time**	0.36	.55	0.48	.49	0.34	.56	0.02	.89	0.11	.74	0.04	.84
**CS:Region**	2.36	.12	0.002	.96	0.001	.97	1.25	.26	0.01	.93	0.15	.70
**Time:Region**	9.39*	**.002**	116.01***	**<.0001**	45.61***	**<.0001**	0.001	.97	0.80	.37	0.001	.99
**CS:Time:Region**	0.01	.93	0.14	.71	0.08	.78	0.001	.97	0.13	.71	0.04	.85

*Note*. The asterisks denote the level of significance, also denoted in the *p* values. Bold entries with no asterisks denote results that were no longer significant after correcting for multiple comparisons.

Abbreviation: CS, conditioned stimuli.

In contrast, gamma power was not different for CS+/CS− during maintenance. Low gamma power decreased over time, (Table 1, Figure 6b), while high gamma power increased in amygdala but not hippocampus over time (Figure 6). Importantly, these effects of time are unlikely to reflect fatigue/habituation, because they were much less pronounced during the extinction phase. The only effect of time during the extinction phase occurred for the high gamma frequency band, but this was not significant after correcting for multiple comparisons (*F*(1,7984) = 5.96, *p*_uncorr_ = .02).

### 3.4 Neural synchrony between the hippocampus and amygdala

**Figure 7:**
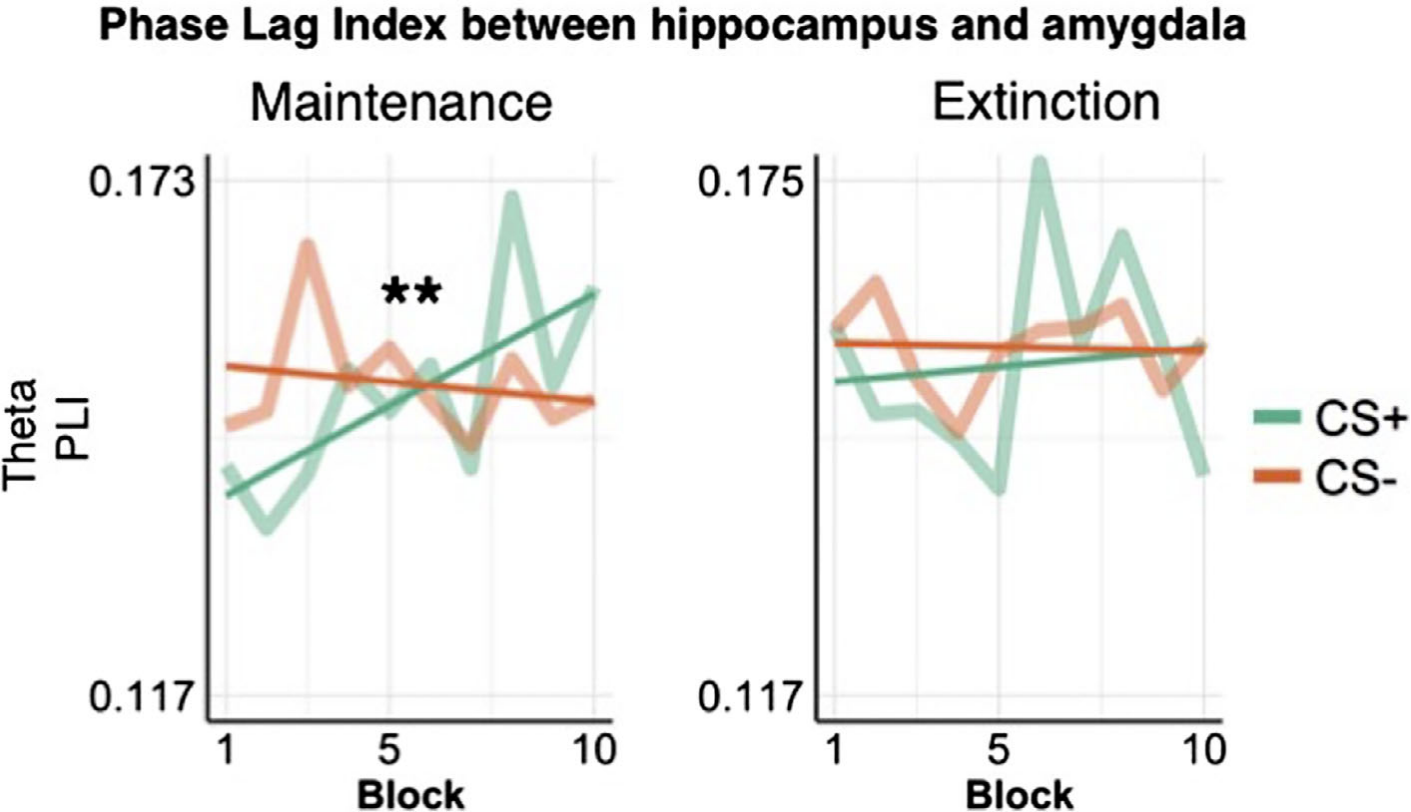
PLI between the hippocampus and amygdala across experimental blocks. The PLI showed a time by CS interaction during the maintenance phase only (*F*(1,3916) = 5.23, *p* = .02), due to an increase in neural synchrony in response to CS+ trials over the time course of the experiment. Straight lines represent a linear fit of the PLI over time. CS, conditioned stimuli; PLI, phase lag index [Color figure can be viewed at http://wileyonlinelibrary.com]

We next assessed neural synchrony between the hippocampus and amygdala using the PLI (Stam et al., 2007), in the theta frequency band. For this analysis, we focused on the theta band, as it typically underlies threat learning in rodents (Lesting et al., 2013). There was no main effect of CS on PLI in the maintenance and in the extinction phase (Table 2). We observed a main effect of time and a significant time by CS interaction in the theta band during the maintenance phase (Table 2). Post hoc analyses showed that the PLI for CS+ trials significantly increased over the time‐course of the experiment (Figure 7, *F*(1,1960) = 7.49, *p* = .006), while it did not differ across time for CS− trials (*F*(1,1952) = 0.23, *p* = .63) (Figure 7).

**Table 2:** Statistical analysis of the PLI between the hippocampus and amygdala in theta band, for maintenance and extinction phases

	Maintenance		Extinction	
	*F*(1,3916)	*p*‐Value	*F*(1,3990)	*p*‐Value
CS	0.08	.77	0.29	.59
Time	2.44	.12	0.07	.79
CS:Time	5.23	.02	0.19	.66

*Note*. Bold highlights significant results.

Abbreviations: CS, conditioned stimuli; PLI, phase lag index.

## 4 DISCUSSION

We used high‐precision MEG in combination with generative models of participant‐specific anatomical information to characterise, for the first time, amygdala/hippocampal oscillations during human fear conditioning, and to disentangle the underlying neural sources. We showed that including neural sources in hippocampus and amygdala explained our data significantly better than an exclusively neocortical set of sources. Moreover, we showed that these models were highly sensitive to the underlying single‐participant brain anatomy, as displacing these deep structures by only a few millimetres resulted in a significant drop in model evidence.

Importantly, using high precision MEG recordings, we demonstrate that theta, but not gamma, oscillations in the hippocampus and amygdala relate to predicted threat, in accordance with previous rodent studies (Seidenbecher et al., 2003; Stujenske et al., 2014). Notably, however, the direction of the CS+/CS− difference was in the opposite direction compared to what is observed in rodents, which we discuss below. In anticipation of an aversive event, theta power in the hippocampus and amygdala was decreased compared to safety, while neural synchrony between these two regions increased over time, as more predictors of threatening events were observed. Gamma oscillations did not relate to an upcoming threat per se, but high gamma (70–120 Hz) power in the amygdala increased over trial repetitions during maintenance, a finding that accords with reports in rodents (Stujenske et al., 2014). Theta power was lower for CS+ than CS− also during the extinction phase, at least 24 h after the last US exposure, although this finding was not significant after correction for multiple comparisons.

### 4.1 High‐precision MEG at the single‐participant level

Neuroimaging studies generally rely on a larger number of participants (N = 20), but include relatively few stimulus repetitions for each of them (e.g., 116 CS± acquisition and extinction trials in (Lithari et al., 2015), or 40 CS± acquisition trials in (Chien et al., 2017)). Here, we opted for a different approach, including few participants (*N* = 5), but increasing the precision of single measurements for each one of them (Meyer, Bonaiuto, et al., 2017; Troebinger, López, Lutti, Bradbury, et al., 2014), akin to what used in the field of precision functional mapping (Gordon et al., 2017) and the nonhuman primate literature (Klavir et al., 2013). As we were interested in fine‐grained spatial information, we built participant‐specific generative models, accounting explicitly for interindividual differences in brain anatomy. Moreover, we increased the SNR of each single measurement in two ways: first, we minimised sources of noise due to head movements or coregistration errors by fixing the position of participants' heads during the MEG data acquisition (Supporting Information 1), using flexible headcasts (Meyer, Rossiter, et al., 2017; Troebinger, López, Lutti, Bradbury, et al., 2014); second, the use of headcasts allowed us to build up large numbers of trials per participant (*N* = 800 trials) over separate recording sessions.

Previous studies, based on simulations, have shown the theoretical potential of this technique to localise neural oscillations from the hippocampus (Meyer, Rossiter, et al., 2017), and to distinguish responses originating between deep and superficial cortical layers (Troebinger, Lopez, Lutti, Bestmann, et al., 2014). However, none of these studies has confirmed these simulation results in human MEG data. Here, we built upon previous theoretical work and provided, for the first time, experimental evidence that subcortical oscillations can be reconstructed using headcast‐based MEG recordings with a spatial resolution of few millimetres. Moreover, for our main analyses, we have used LME models, which take full advantage of single‐trial repetitions and include participants as random effects, instead of requiring a trial averaging.

We sought to reconstruct oscillatory activity not only from the hippocampus (Meyer, Rossiter, et al., 2017), but also from the amygdala. Although the amygdala is a small structure, with a volume of ~1 cm^3^, located relatively far from the sensors (~8–9 cm), its neuronal density is up to six times higher than the cortex (Dumas et al., 2013; Pakkenberg & Gundersen, 1997; Schumann & Amaral, 2005). Previous studies have shown that, because of this elevated neuronal density, only 0.2–0.3 cm^3^ of activated current would be sufficient to generate a magnetic signal that can be captured by MEG sensors, even if the cells are not pyramidal and not aligned in parallel (Dumas et al., 2011, 2013). Here, we built upon previous studies which have demonstrated the feasibility of reconstructing activity from the amygdala, in healthy conditions (Attal et al., 2012; Dumas et al., 2013), or during epileptic discharges (Pizzo et al., 2019) and show that a model containing amygdala sources is significantly more likely to describe MEG data at the sensor level compared to a model with cortical or cortical and hippocampal sources alone, despite identical model complexity.

### 4.2 Neural responses in the hippocampus and amygdala during maintenance and extinction of fear memories

The neural circuitry of threat conditioning has been well studied in rodents, with a large literature providing converging evidence that the amygdala and hippocampus are key structures in differentiating safety versus threat (Adhikari et al., 2015; Lesting et al., 2013; Narayanan et al., 2007; Paz et al., 2008; Seidenbecher et al., 2003; Stujenske et al., 2014). Theta oscillations and neural synchrony are pronounced in the rodent amygdala and hippocampus during presentation of cues encoding threat (presentation of CS+ stimuli) (Seidenbecher et al., 2003; Stujenske et al., 2014) and decrease during states of relative safety, possibly as a result of high frequency modulatory oscillatory input from the medial prefrontal cortex (Lesting et al., 2013; Likhtik & Paz, 2015; Stujenske et al., 2014).

In humans, it has been more difficult to study the role of the amygdala in acquisition and extinction of fear memories. There is no meta‐analytic evidence that overall hemodynamic responses in amygdala relate to CS+/CS− (Fullana et al., 2015), possibly due to the sparse distribution of CS+‐responsive neurons (Reijmers, Perkins, Matsuo, & Mayford, 2007) and the large population of neurons that respond to CS+ absence (Ciocchi et al., 2010). Patterns of evoked hemodynamic responses have been reported to differ between CS+ and CS− (Bach, Weiskopf, & Dolan, 2011; Staib & Bach, 2018; Visser, Scholte, Beemsterboer, & Kindt, 2013), in line with rodent optical imaging findings (Grewe et al., 2017). Beyond the amygdala, anticipation of aversive stimuli elicits responses across a distributed network of cortical regions, including the cingulate cortex, insula, dorsolateral prefrontal cortex, or hypothalamus (Fullana et al., 2015), suggesting that the neural circuit underlying threat conditioning in humans may be modulated by cognitive influences and thus have a more complex architecture than that of rodents (Janak & Tye, 2015).

### 4.3 Measuring neural oscillations during threat conditioning in humans

Despite the large number of hemodynamic studies on threat conditioning in humans, there is still little information about its oscillatory content and thus its dynamic neural implementation. As in our case, previous studies investigating evoked amygdala responses using EEG/MEG (Balderston et al., 2014; Moses et al., 2007) had to deal with the ill‐posed problem of reconstructing source responses from sensor‐level MEG or EEG data, which is amplified by the low SNR of responses originating far from the sensors. To corroborate their conclusions, Balderston et al. (2014) analysed the signal from nearby temporal cortex to demonstrate that it has a different structure. This is an interesting approach, albeit limited to selected regions of interest, and relying on the same source reconstruction as the extraction of the amygdala signal in the first place.

Here, we used headcasts to maximise SNR in order to get a precise estimate of a participant's anatomy with respect to the MEG sensors (Meyer, Bonaiuto, et al., 2017). Maximal SNR, minimal coregistration error, and the MSP algorithm as used here have been shown to be optimal in identifying the exclusive contribution of deep cortical structures (such as the hippocampus) to MEG data (Meyer, Rossiter, et al., 2017). Although we cannot exclude the possibility that reconstructed responses from amygdala might be, at least to some extent, mixed with responses from the hippocampus (and vice versa), we provide quantitative evidence that explicitly modelling sources in the amygdala significantly improves the likelihood of observing the recorded MEG data at the sensor level, over and above modelling cortical or cortical and hippocampal sources. Importantly, by imposing sparsity, we could refine the spatial precision of our findings by showing that even displacements of 3 mm are enough to significantly deteriorate the likelihood of our generative models. This finding is in accordance with recent studies showing that sparsity is required to extract fine‐grained information on the underlying sources (Bonaiuto, Rossiter, et al., 2018; Krishnaswamy et al., 2017).

Our findings largely accord with what previous MEG/EEG studies have reported for the sensor level or for cortical areas. In these, CS+ presentations have been shown to result in a decrease in the power of slow oscillations, compared to presentations of CS− (Chien et al., 2017; Lithari et al., 2016; Moses et al., 2010). This pattern of results was replicated in our study, where theta power in the hippocampus and amygdala was significantly lower following presentations of CS+ compared to CS−. This result is the opposite of what rodent studies have reported (Seidenbecher et al., 2003; Stujenske et al., 2014), and it has been interpreted as an anticipatory mechanism, which in humans results in a suppression of ongoing neural activity and higher excitability in light of an upcoming aversive event (Lithari et al., 2016). Increase in neural excitability has been previously reported in humans, in anticipation of predicted stimuli in different sensory modalities and cortical areas (Langner et al., 2011), and in particular in response to aversive stimuli (Lithari et al., 2016; Ploghaus et al., 1999). An alternative interpretation of amygdalar responses in our experimental paradigm is that the absence of a US in CS− trials acts as a reward, and thus a higher oscillatory power in response to CS− compared to CS+ could be explained by amygdalar responses in encoding rewards (Murray, 2007; Peck & Salzman, 2014). Indeed, this interpretation follows the view that extinction learning is in fact, reward learning (Janak & Tye, 2015), and that distinct populations of amygdalar neurons contribute to learning and extinction of fear memories (Muller et al., 2008).

A further difference between our study and the majority of studies in rodents is that the latter typically preselect those animals who have successfully learned CS–US associations, as assessed through behavioural measures, such as measuring the amount of time spent freezing in response to CS+ compared to CS−, and then splitting animals according to whether they froze for a higher amount of time for CS+ versus CS−, likely boosting the significance of findings (Likhtik, Stujenske, Topiwala, Harris, & Gordon, 2014; Stujenske et al., 2014). This prior selection singles out effects of successful conditioning, but relies on heterogeneous criteria for defining which animal was conditioned and which was not. Another major difference is that the US, we used in this study (i.e., loud sounds) are unlikely to induce the same degree of aversion and protective responses as electric shocks in rodents.

As a limitation, our strategy of maximising SNR at the single‐participant level mandated a large number of stimulus repetitions, in line with typical nonhuman primate electrophysiology but different from many human and rodent studies, which include more participants with fewer trial repetitions. We note that there is no evidence that electrophysiological responses to CS+ and CS− decrease over time in rodents or humans during fear maintenance after initial overtraining. Equally, a large meta‐analysis has not found evidence that differential hemodynamic responses to CS+ and CS− in humans decay over time (Fullana et al., 2015). In contrast, the discriminability between hemodynamic response patterns evoked by CS+ and CS− has been shown to increase over 160 trials (Bach, Weiskopf, & Dolan, 2011), suggesting that there is no a priori reason to expect that our results are incomparable to those obtained with fewer trial repetitions in rodents or humans.

In summary, we use high‐precision MEG to show, for the first time, that the power of theta oscillations in the human hippocampus and amygdala are decreased in anticipation of upcoming threat compared to safety, while neural synchrony between these two regions increases as associations between neutral events and upcoming threat are learnt.

5

### DATA AVAILABILITY

The data that support the findings of this study are available from the corresponding author upon reasonable request.

## Supporting information


**Appendix S1** Supporting information
