# Supplementary material for: High‐precision magnetoencephalography for reconstructing amygdalar and hippocampal oscillations during prediction of safety and threat
Source: Hum Brain Mapp. 2019 Jun 30;40(14):4114–29. doi: 10.1002/hbm.24689 (PMC6772181; doi:10.1002/hbm.24689)
Supplement: Supplementary file 1 — Appendix S1 Supporting information [file HBM-40-4114-s001.pdf]

Supporting Information for:

High-precision magnetoencephalography for reconstructing amygdalar and hippocampal oscillations during prediction of safety and threat

Tzovara A., Meyer S.S., Bonaiuto J.J., Abivardi A., Dolan R.J., Barnes G.R., Bach D.R.

Supporting Information 1: Head motion during the experiment

To assess the level of head motion during our MEG recordings we extracted the time-courses of the locations of the fiducial coils for each participant and session. The time-courses of coil locations were mean corrected per session to obtain relative estimates of head movement along three dimensions (x,y,z), as in previous studies (Meyer, Bonaiuto, et al. 2017).

The mean time-courses of coil movement across participants for each of the 10 experimental sessions of maintenance and extinction phases are displayed in Supplemental Figure 1. For the X and Y dimensions the mean within session head movement across participants was bellow 0.5 mm for all coils/time points /sessions (Supplemental Figure 1). In accordance with previous studies (Meyer, Bonaiuto, et al. 2017), the maximum coil movement was observed for the Z dimension (upwards/downwards movements) and was similar for all three coils (LPA, RPA and nasion).

Next, we evaluated the average within session standard deviation of coil displacement for all dimensions and coils. For the maintenance phase the within session standard deviation of average head movement was less than 0.26, 0.21 and 0.32 mm for the LPA, RPA and nasion, respectively, and for the extinction phase less than 0.15, 0.13 and 0.22 mm.

Importantly, head movement was similar for maintenance and extinction phases, suggesting that the weaker difference in theta oscillations observed during extinction is not due to a lower signal-to-noise ratio.

Supporting Information 2: Source reconstruction using Empirical Bayes Beamformer (EBB) algorithm

In all of our main analyses we used a Multiple Sparse Priors (MSP) algorithm, due to its suitability for identifying activity from subcortical sources (Meyer, Rossiter, et al. 2017). We replicated our findings and model selection results using Empirical Bayes Beamfomer (EBB; (Belardinelli et al. 2012; Lopez et al. 2014)), which consists of optimizing a single covariance prior at the source level, which is estimated directly from the data.

We applied EBB in the same data as we did for MSP: we considered single-trials from 0 – 3500 ms, relative to the CS onset, for both experimental conditions, and a broadband signal of 1 – 120 Hz. The total number of solution points is the same as the number of vertices in the anatomical models for each participant, which for the cortical model are 8196 for all participants, for model A 8253 ± 1, for model H 8676 ± 15 and for model HA 8732 ± 15 (mean across participants ± standard error).

Using EBB confirmed the results that we had with MSP: adding hippocampal and amygdalar meshes resulted in a significant increase in Free Energy (F) values (Supplemental Figure 2). The increase in F values relative to a baseline model, containing a cortical mesh only was in all cases greater or equal to 263, suggesting that activity from these regions significantly contributes in the observed MEG data

at the sensor level (Supplemental Figure 2). The increase in F values when considering the full model was much higher with EBB compared to MSP (Figure 3 of the main text). The most likely reason is that whereas MSP relies on an exhaustive search of possible patches in order to establish an optimal source distribution - and is therefore prominent to local extrema - EBB makes use of a single empirical source distribution and is computationally more robust (although participant to more assumptions about the source distribution). Although it should be noted that there is a small increase in the complexity of the underlying source distribution (adding the subcortical structures increases the number of vertices increases by 1%) when using EBB (which is controlled for in MSP by keeping the number of priors constant) our simulation studies (Meyer et al., 2017) do not suggest that this should bias the method.

Nevertheless, we performed an additional control analysis on the full model, containing hippocampal, amygdalar and cortical meshes (model HA), by keeping the complexity of the anatomical model constant and displacing the hippocampal and amygdalar meshes along the anterior/posterior and inferior/superior axes, in a similar way as done with MSP (Figure 3). Results of this analysis showed that the more we displace these meshes, the larger the decrease in F values (Supplemental Figure 2). Nevertheless, this displacement needs to be of at least 20 mm to result to a significant decrease in F values across participants. We remind the reader that in the case of MSP, a displacement of 3 mm was enough to result in similar amounts of drop in F values (Figure 3).

These control analyses show that both EBB and MSP provide converging evidence that considering activity from the hippocampus/amygdala in our generative models improves the description of the MEG responses observed at the sensor level, suggesting that both of these regions are actively engaged during our experimental manipulations. The low spatial specificity of the EBB compared to MSP, supports the sensitivity of sparse algorithms to a precise localization of subcortical sources, which is in accordance with previous studies (Meyer, Rossiter, et al. 2017).

Supporting Information 3. Single participant analysis

A. Model selection for single participants

In addition to group analyses, we evaluated the robustness of our findings at the single participant level. First, we repeated model selection results for each participant separately. With MSP, adding priors in the hippocampus and/or amygdala resulted in a decisively better model for all participants but one (P3, Supplemental Figure 3). For P3, adding sources in the amygdala, or amygdala and hippocampus the model evidence remained the same as that of the model with cortical sources only (change in free energy values, $\Delta F < 3$). With EBB (right panel), adding amygdala and hippocampal sources resulted in a decisive increase in model evidence for all participants, compared to the cortical model only (change in free energy values, $\Delta F > 3$). These results reinforce the group level findings, based on fixed effects analysis (Figure 3a of main text) by demonstrating that adding sources in the hippocampus and/or amygdala results in a decisive increase in model evidence compared to models with sources in the cortex only, irrespective of the algorithm used for model inversion.

B. Single participant theta power

We examined whether the main finding of our manuscript, and namely the suppression in theta power for CS+ compared to CS- stimuli, can be observed at the single-participant level. Similar as for the group, we computed the power of the time-course of sources located in the hippocampus/amygdala. Visual inspection of the data confirmed the results that we obtained at the group level, and namely that during threat (CS+), theta power is lower than during safety (CS-), and that this reduction is more evident in the maintenance phase (Supplemental Figure 4).

We used linear mixed effects models (LMEs), to evaluate whether the difference observed between CS+/- stimuli was significant for single participants. We used a similar formula as for the group level, with fixed effects of CS, time and region. For the case of single participant LMEs we considered random factors of time instead of participants. Results of this comparison confirmed the group results for two participants out of five, with a significant main effect of CS (Supplemental Table 1, P1 and P5). For the remaining participants, we observed a non-significant trend into the same direction for 4/5 participants in the amygdala, and for 3/5 participants in the hippocampus during the maintenance phase (Supplemental Figure 4). During extinction, a main effect of CS was observed for one participant (P1), and theta power was lower, but not significantly lower, for CS+ compared to CS- trials for 1/5 and 2/5 additional participants in the amygdala and hippocampus, respectively. Moreover, consistent to group results, all participants showed a main effect of time during maintenance, but not during extinction, where only one participant showed a weak effect of time (Supplemental Table 1).

Supporting Information 4: Selection of linear mixed effects model

In order to statistically contrast oscillatory power in different frequency bands, we computed linear mixed effects (LME) models, using package 'nlme' in R (www.r-project.org; version 3.2). We considered four factors that depended on the experimental design: CS (CS+/-), Region (amygdala/hippocampus) and Time (measured in blocks), and an additional fourth factor of participant, to account for inter-participant variance.

To avoid inflation of our statistical effects (Westfall et al. 2017) we considered several LME models, all of which had a random effect of 'participant' and fixed effects of all the other three factors (i.e. CS, Region, Time) and additionally random effects for some or none of these factors (Supplemental Table 2 for an overview of the LME model space). We selected the LME model based on Akaike Information Criterion (AIC (Penny et al. 2004)). Importantly, this model selection was blind to the contrast of interest for our main analyses, as it was only informative of the likelihood of observing the reconstructed MEG oscillatory power in the hippocampus and amygdala, given the models. We considered broadband responses for this analysis, at 1-120 Hz, the same range as the one used for the source reconstruction techniques, and then used the winning model for all subsequent analyses, when decomposing responses to specific frequency bands.

The model with the lowest AIC value was the one including CS, Block, Region as Fixed factors, Participants as Random factors and additionally including a separate intercept for Region (Model 2), corresponding to a difference in AIC values of -2989.63 with respect to Model 1 (Supplemental Table 2).

Supporting Information 5: The influence of the number of priors on source reconstruction

We evaluated how the total number of priors included in the MSP algorithm influences its sensitivity in reconstructing deep sources. For our main analyses we opted for a quite low number of priors (N = 100 priors), to sparsely cover the whole brain, as we were interested in characterizing discrete sources only. We additionally examined whether the inclusion of deep sources would benefit a less sparse implementation of MSP, including 500 priors, a number that is typically used in previous studies. Similar as with 100 priors, we constructed four different models, including either all 500 priors on the cortex (model C), or 490 priors on the cortical and 10 on the hippocampal, or amygdalar meshes (models H and A, respectively), or 480 priors on the cortical, 10 on the hippocampal and 10 on the amygdalar meshes (model HA).

We found that even in this case of a much denser cortical coverage, the inclusion of sources in the hippocampus and/or amygdala results in a significantly better explanation of MEG data recorded at the sensor level, indexed by a significant increase in model evidence for all models (FHA-FC = 13.44, FHA-FC = 15.45, FHH-FC = 14.88, Supplemental Figure 5). Although these is still a clear advantage in including subcortical sources, this advantage in the case of 500 priors is smaller than in the case of 100, as the inclusion of ~x5 times more cortical sources might obscure some of the signal of subcortical sources.

Supporting Information 6: Model selection results for maintenance and extinction

We expected that our current experimental setup would elicit the strongest activation of hippocampal and amygdalar sources during the maintenance phase, while CS-US associations are still reinforced. We thus used trials of the maintenance phase only to examine whether sources from the hippocampus & amygdala are needed to explain MEG data at the sensor level (Figure 3 of the main text). For reasons of completeness, we repeated the model comparison using trials of the extinction phase. Similar as with maintenance, the full model, containing hippocampal, amygdalar and cortical sources gave the highest model evidence (Supplemental Figure 6, model HA). However, in contrast to maintenance, model HA missed the threshold for significance with respect to models with cortical sources only (F_{AH}-F_{C} = 2.87). Models with amygdalar or hippocampal sources only had significantly lower model evidence (F_{HA}-F_{C} = -3.97, F_{H}-F_{C} = -5.47) compared to models C and HA. These results suggest that the overperformance of MSP models with hippocampal and amygdalar priors is specific to a case where amygdalar and hippocampal sources are strongly activated (i.e. maintenance of threat memories), and not just a generic property of the source localization algorithm.

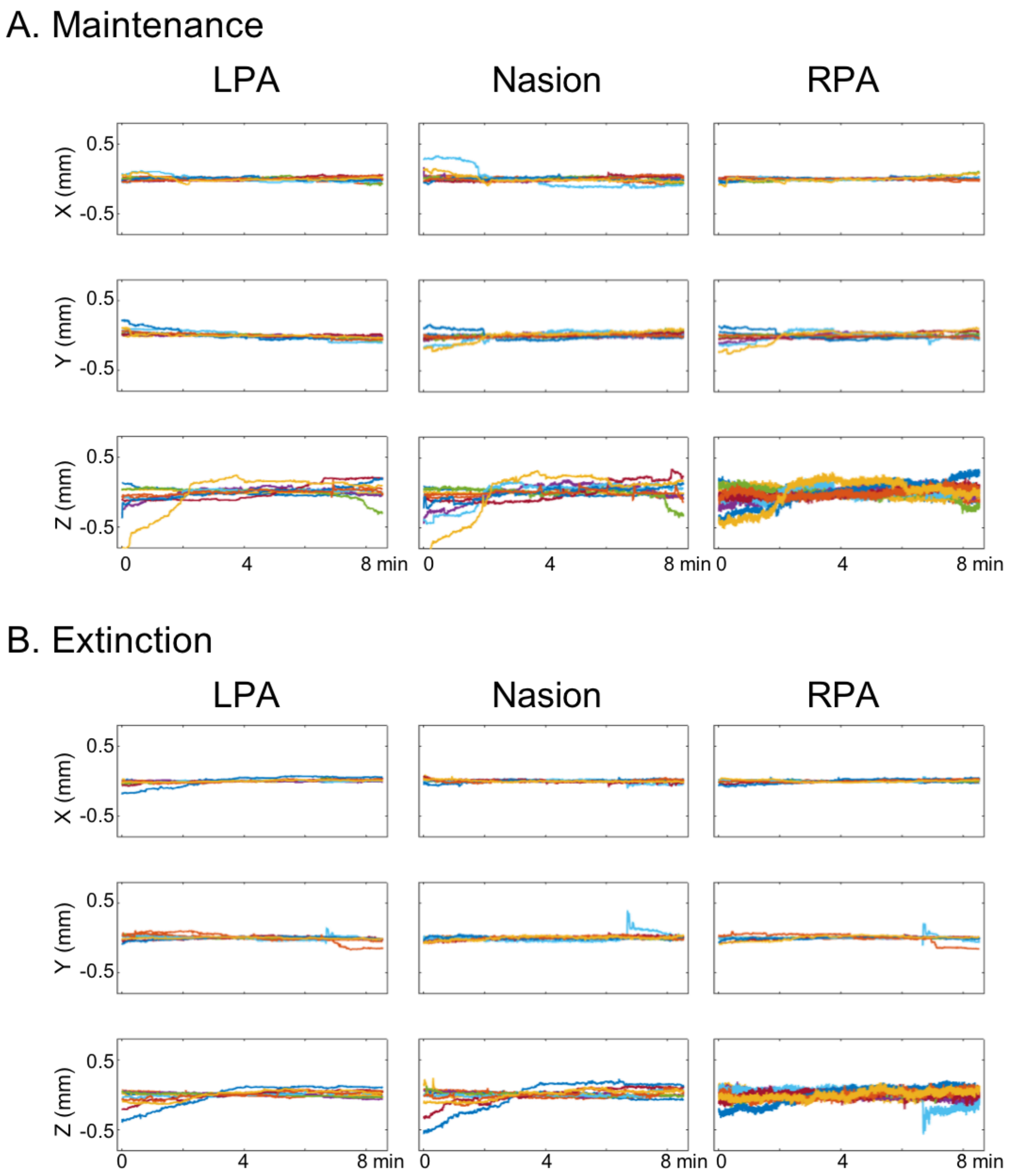

Supplemental Figure 1. Within session head movement for maintenance (a) and extinction (b) phases across three fiducial locations (LPA, nasion and RPA). Each line corresponds to the time-course of coil displacement for a given session, averaged across participants. In total there were 10 sessions per participant and experimental phase.

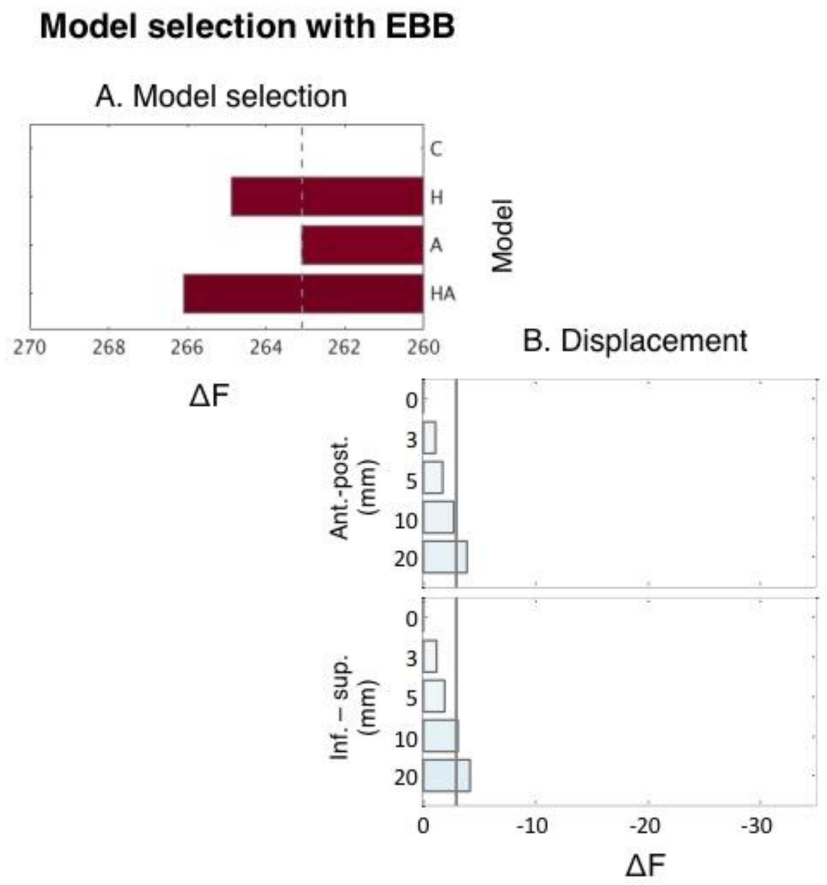

Supplemental Figure 2. (a) Model selection results using EBB, highlighted by an increase in Free Energy by adding hippocampal (H) and amygdaladar (A) meshes to a cortical model only. (b/c) Free Energy decreases as we displace the hippocampal/amygdalar meshes, but this decrease is significant only when the displacement is at least 20 mm, suggesting a lower spatial sensitivity for EBB compared to MSP (Figure 3 of main manuscript).

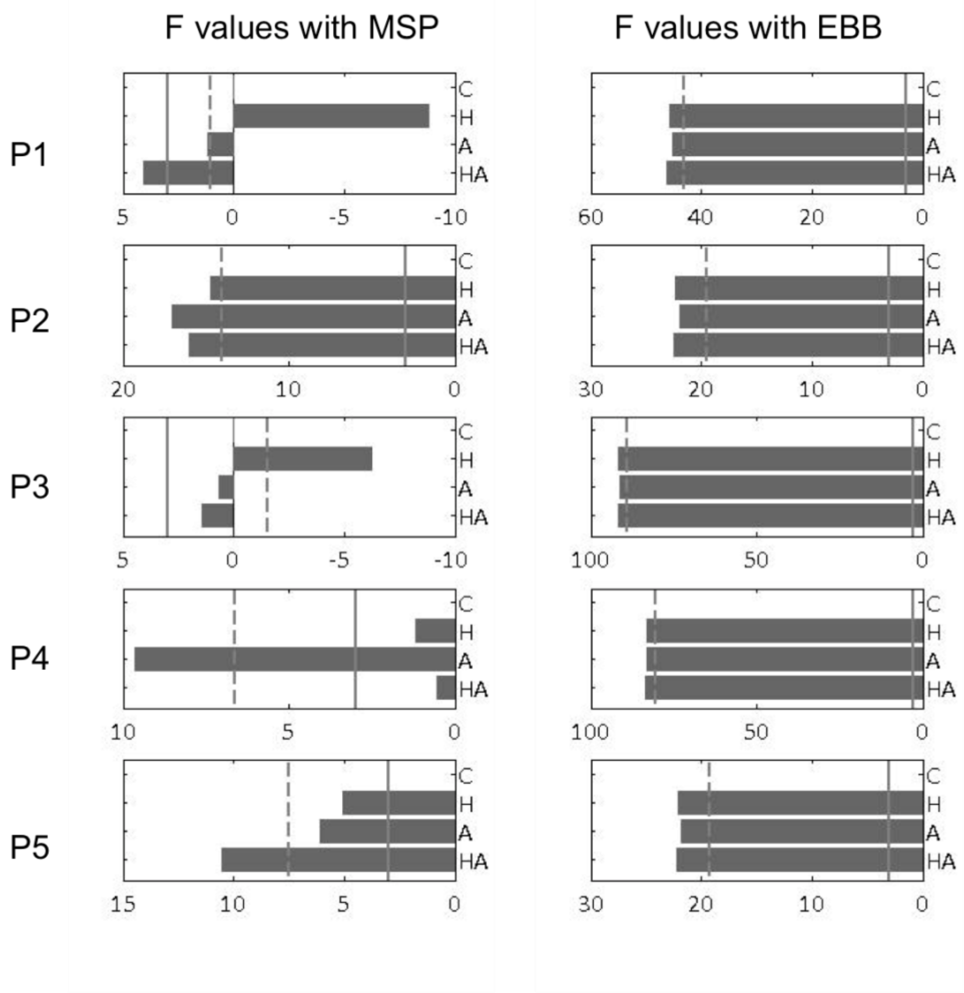

Supplemental Figure 3. Model selection for single participants. Bars illustrate free energy (F) values for each model, relative to the cortical model only. Full lines illustrate the critical threshold, above which models perform significantly better compared to a model containing cortical priors only (model C).

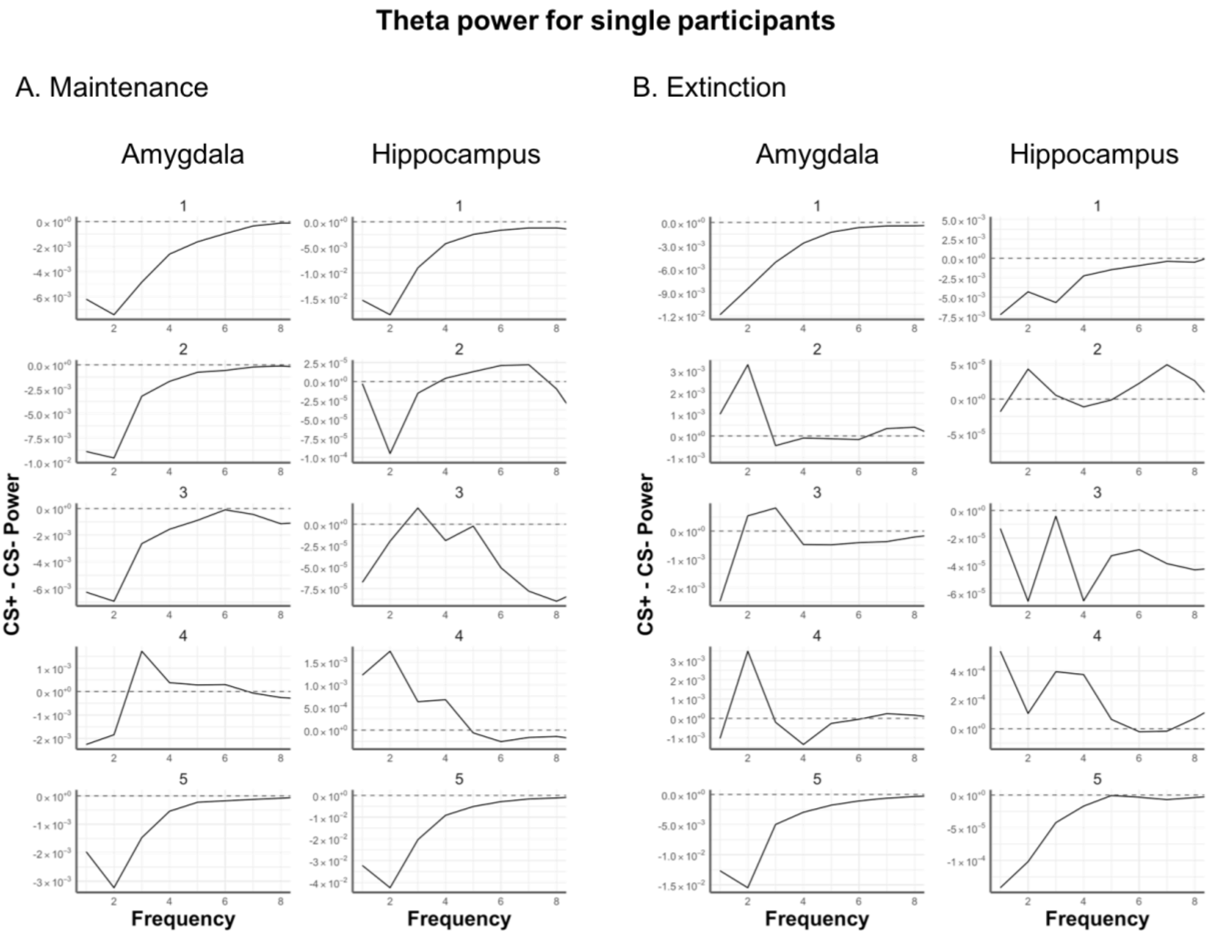

Supplemental Figure 4. Single-participant difference in mean theta power for CS+ minus CS- trials in the hippocampus and amygdala for maintenance (panel a) and extinction (panel b) phases. The horizontal dashed lines denote a difference of zero. During maintenance, theta power was lower in response to CS+ compared to CS- for 4/5 participants in the amygdala, and for 3/5 participants in the hippocampus. During extinction a lower CS+ power was observed for 2/5 and 3/5 participants in the amygdala and hippocampus, respectively.

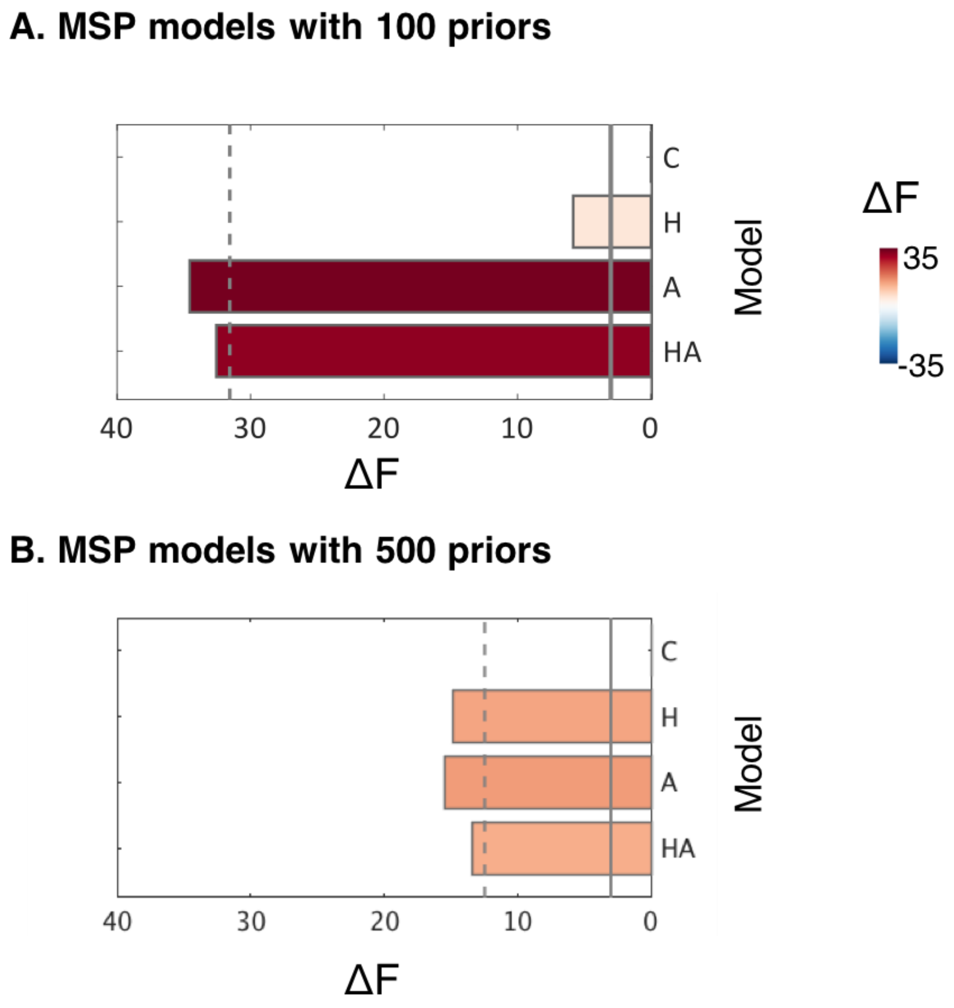

Supplemental Figure 5. Model selection results for MSP while changing the total number of priors, from 100 to 500 priors. In both cases, model evidence significantly increases by including more priors in the hippocampus and amygdala. The increase is more striking with the sparser implementation of MSP (N=100 priors). Full lines denote the threshold for significant model increase with respect to the baseline cortical model, dashed lines the threshold for significant model decrease with respect to the model with the highest model evidence.

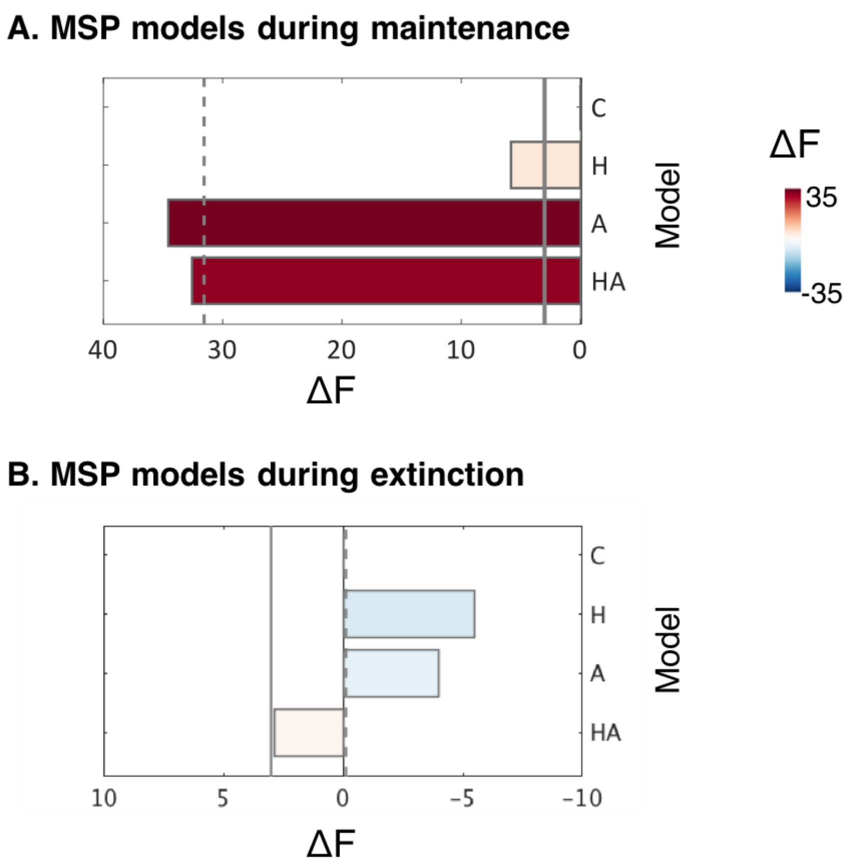

Supplemental Figure 6. Model selection results for maintenance and extinction. During maintenance the inclusion of amygdalar and hippocampal priors results in a significantly higher model evidence, but this is not the case during extinction, possibly due to a less strong oscillatory power originating from these two structures. This result highlights that the increase in model evidence due to the inclusion of hippocampal and amygdalar sources is specific to a case where these sources are strongly activated, and not an artefact due to the anatomy of the forward model.

Supplemental Table 1. Results of linear mixed effects models for single participants (numbered as columns), in theta range, during maintenance (a) and extinction (b) phases.

A. Theta power, maintenance phase

	1		2		3		4		5	
	F	p	F	p	F	p	F	p	F	p
**CS**	**29.36**	**0.000**	2.40	0.121	0.75	0.387	0.00	0.979	**15.91**	**0.000**
**Time**	0.74	0.413	10.05	0.013	1.41	0.268	0.10	0.758	3.08	0.117
**Region**	**807.58**	**0.000**	**425.03**	**0.000**	**498.42**	**0.000**	**221.50**	**0.000**	**692.45**	**0.000**
**CS:Time**	0.02	0.879	0.74	0.390	0.36	0.549	0.38	0.537	0.20	0.651
**CS:Region**	**4.20**	**0.041**	2.39	0.122	0.71	0.401	0.02	0.884	**12.12**	**0.001**
**Time:Region**	**4.89**	**0.027**	**27.18**	**0.000**	2.93	0.087	0.03	0.856	**33.42**	**0.000**
**Time:Region:CS**	0.56	0.456	0.69	0.407	0.37	0.545	0.27	0.603	0.20	0.656

B. Theta power, extinction phase

	1		2		3		4		5	
	F	p	F	p	F	p	F	p	F	p
**CS**	**12.36**	**0.000**	0.05	0.818	0.10	0.748	0.04	0.841	1.84	0.175
**Time**	0.00	0.998	**5.72**	**0.044**	0.39	0.552	1.10	0.325	4.47	0.067
**Region**	**486.60**	**0.000**	**736.65**	**0.000**	**303.13**	**0.000**	**2061.23**	**0.000**	**465.29**	**0.000**
**CS:Time**	**4.93**	**0.027**	0.00	0.956	0.37	0.544	0.04	0.847	1.23	0.268
**CS:Region**	0.28	0.597	0.05	0.827	0.07	0.790	0.00	0.967	1.81	0.178
**Time:Region**	0.02	0.885	**20.22**	**0.000**	0.28	0.599	1.96	0.162	**11.66**	**0.001**
**Time:Region:CS**	2.51	0.113	0.01	0.944	0.32	0.573	0.00	0.970	1.20	0.274

Supplemental Table 2. Selection of a linear mixed effects model.

**Model**	**Fixed effects**	**Random effects**	**AIC value**
1	CS, Block, Region	Participant	0
2	CS, Block, Region	Participant, Region	-2989.63
3	CS, Block, Region	Participant, CS	3.91
4	CS, Block, Region	Participant, Block	1.25

Supplemental References

Belardinelli P, Ortiz E, Barnes G, Noppeney U, Preissl H. 2012. Source Reconstruction Accuracy of MEG and EEG Bayesian Inversion Approaches. PLoS One. 7.

Lopez JD, Litvak V, Espinosa JJ, Friston K, Barnes GR. 2014. Algorithmic procedures for Bayesian MEG/EEG source reconstruction in SPM. Neuroimage. 84:476–487.

Meyer SS, Bonaiuto J, Lim M, Rossiter H, Waters S, Bradbury D, Bestmann S, Brookes M, Callaghan MF, Weiskopf N, Barnes GR. 2017. Flexible head-casts for high spatial precision MEG. J Neurosci Methods. 276:38–45.

Meyer SS, Rossiter H, Brookes MJ, Woolrich MW, Bestmann S, Barnes GR. 2017. Using generative models to make probabilistic statements about hippocampal engagement in MEG. Neuroimage. 149:468–482.

Penny WD, Stephan KE, Mechelli A, Friston KJ. 2004. Comparing dynamic causal models. Neuroimage. 22:1157–1172.

Westfall J, Nichols TE, Yarkoni T. 2017. Fixing the stimulus-as-fixed-effect fallacy in task fMRI. Wellcome Open Res. 1:1:23.
